# Supplementary material for: Hybrid Models and Biological Model Reduction with PyDSTool
Source: PLoS Comput Biol. 2012 Aug 9;8(8):e1002628. doi: 10.1371/journal.pcbi.1002628 (PMC3415397; doi:10.1371/journal.pcbi.1002628)
Supplement: Text S4 — Complete source code for the PyDSTool package (version 0.88.120504). Includes API documentation and help files linking to web pages. This file is identical to the current public release on Sourceforge.net. (ZIP) [file pcbi.1002628.s004.zip › PyDSTool/html/PyDSTool.PyCont.Continuation-pysrc.html]

xml version="1.0" encoding="ascii"?


PyDSTool.PyCont.Continuation


| Home | Trees | Indices | Help | PyDSTool |
| --- | --- | --- | --- | --- |

Package PyDSTool ::
Package PyCont ::
Module Continuation

[hide private]

[frames] | no frames]

# Source Code for Module PyDSTool.PyCont.Continuation

```
   1  """ Curve classes: Continuation, EquilibriumCurve, FoldCurve, HopfCurveOne, HopfCurveTwo
 
   2  
 
   3      Drew LaMar, March 2006
 
   4  
 
   5      Continuation is the ancestral class of all curve classes and contains the continuation algorithms
 
   6      (Moore-Penrose, etc.)  It also contains all methods that are general to any curve found using
 
   7      continuation.
 
   8  """ 
   9  
 
  10  # -----------------------------------------------------------------------------------------
 
  11  
 
  12  from misc import * 
  13  from TestFunc import * 
  14  from BifPoint import * 
  15  from Plotting import * 
  16  
 
  17  from PyDSTool import Point, Pointset, PointInfo, args 
  18  from PyDSTool.common import pickle, sortedDictValues, sortedDictKeys 
  19  from PyDSTool.errors import * 
  20  from PyDSTool.Symbolic import QuantSpec 
  21  try: 
  22      from PyDSTool.matplotlib_import import * 
  23  except ImportError: 
  24      from PyDSTool.matplotlib_unavailable import * 
  25      print "Warning: matplotlib failed to import properly and so is not" 
  26      print "  providing a graphing interface" 
  27  
 
  28  from numpy.random import random 
  29  from numpy import dot as matrixmultiply 
  30  from scipy import optimize, linalg 
  31  from numpy import array, float, complex, int, float64, complex64, int32, \
 
  32      zeros, divide, subtract, arange, all, any, argsort, reshape, nonzero, \
 
  33      log10, Inf, NaN, isfinite, r_, c_, sign, mod, mat, \
 
  34      subtract, divide, transpose, eye, real, imag, isnan, resize 
  35  
 
  36  from copy import copy, deepcopy 
  37  from math import ceil 
  38  
 
  39  #####
 
  40  _classes = ['Continuation', 'EquilibriumCurve', 'FoldCurve', 'HopfCurveOne',
 
  41              'HopfCurveTwo', 'FixedPointCurve', 'LimitCycleCurve',
 
  42              'UserDefinedCurve'] 
  43  
 
  44  _constants = ['cont_args_list', 'cont_bif_points', 'equilibrium_args_list',
 
  45                'equilibrium_bif_points', 'fold_args_list', 'fold_bif_points',
 
  46                'hopf_args_list', 'hopf_bif_points', 'limitcycle_args_list',
 
  47                'limitcycle_bif_points', 'fixedpoint_args_list',
 
  48                'fixedpoint_bif_points', 'userdefined_args_list',
 
  49                'all_args_list', 'all_point_types', 'all_curve_types',
 
  50                'bif_curve_colors', 'bif_point_colors',
 
  51                'stab_line_styles','auto_point_types', 'other_special_points',
 
  52                'solution_measures', 'solution_measures_list'] 
  53  
 
  54  __all__ = _classes + _constants 
  55  #####
 
  56  
 
  57  cont_args_list = ['name','force','freepars','MaxNumPoints','MaxCorrIters',
 
  58                    'MaxTestIters','MaxStepSize', 'MinStepSize', 'StepSize',
 
  59                    'VarTol','FuncTol','TestTol', 'description', 'uservars',
 
  60                    'LocBifPoints','verbosity','ClosedCurve','SaveJacobian',
 
  61                    'SaveEigen', 'Corrector', 'UseAuto', 'StopAtPoints',
 
  62                    'SPOut'] 
  63  
 
  64  cont_bif_points = ['BP', 'B', 'SP'] 
  65  
 
  66  equilibrium_args_list = ['LocBifPoints'] 
  67  equilibrium_bif_points = ['LP', 'H'] 
  68  
 
  69  fold_args_list = ['LocBifPoints'] 
  70  fold_bif_points = ['BT', 'ZH', 'CP'] 
  71  
 
  72  hopf_args_list = ['LocBifPoints'] 
  73  hopf_bif_points = ['BT', 'ZH', 'GH', 'DH'] 
  74  
 
  75  fixedpoint_args_list = ['LocBifPoints', 'period'] 
  76  fixedpoint_bif_points = ['PD', 'LPC', 'NS'] 
  77  
 
  78  limitcycle_args_list = ['LocBifPoints', 'NumCollocation', 'NumIntervals',
 
  79                          'AdaptMesh', 'NumSPOut', 'DiagVerbosity',
 
  80                          'SolutionMeasures', 'SaveFlow'] 
  81  limitcycle_bif_points = ['PD', 'LPC', 'NS'] 
  82  
 
  83  userdefined_args_list = ['LocBifPoints'] 
  84  
 
  85  other_special_points = ['RG', 'UZ', 'P', 'MX'] 
  86  
 
  87  auto_point_types = {1: 'BP', 2: 'LP', 3: 'H', 4: 'RG', -4: 'UZ', 5: 'LPC',
 
  88                      6: 'BP', 7: 'PD', 8: 'NS', 9: 'P', -9: 'MX'} 
  89  
 
  90  solution_measures_list = ['max', 'min', 'avg', 'nm2']   # Ordering is important 
  91  solution_measures = dict(zip(solution_measures_list,[0, 0, 1, 2])) 
  92  
 
  93  all_args_list = unique(cont_args_list + equilibrium_args_list + fold_args_list +
 
  94                         hopf_args_list + fixedpoint_args_list +
 
  95                         limitcycle_args_list) 
  96  all_point_types = unique(other_special_points + cont_bif_points +
 
  97                           equilibrium_bif_points + fold_bif_points +
 
  98                           hopf_bif_points + fixedpoint_bif_points +
 
  99                           limitcycle_bif_points) 
 100  all_curve_types = ['EP-C', 'LP-C', 'H-C1', 'H-C2', 'FP-C', 'LC-C'] 
 101  
 
 102  bif_curve_colors = {'EP-C': 'k', 'LP-C': 'r', 'H-C1': 'b', 'H-C2': 'b',
 
 103                      'FP-C': 'g', 'LC-C': 'm', 'UD-C': 'k'} 
 104  bif_point_colors = {'P': 'ok', 'RG': 'ok', 'LP': 'or', 'BP': 'og',
 
 105                      'H': 'ob', 'B': 'dr', 'BT': 'sy', 'ZH': 'sk',
 
 106                      'CP': 'sr', 'GH': 'sb', 'DH': 'sg', 'LPC': 'Dr',
 
 107                      'PD': 'Dg', 'NS': 'Db', 'MX': 'xr', 'UZ': '^r'} 
 108  stab_line_styles = {'S': '-', 'U': '--', 'N': '-.', 'X': ':'} 
 109  
 
 110  
 
 111  
 
 112  
 


113 -class Continuation(object):


114      """Abstract continuation class
 
 115  
 
 116      Children: EquilibriumCurve, FoldCurve, HopfCurveOne, HopfCurveTwo,
 
 117      LimitCycleCurve
 
 118      """ 
 119  
 


120 -    def __init__(self, model, gen, automod, plot, args=None):


121          self.curvetype = args['type'] 
 122          self._ptlabel = self.curvetype.split('-')[0] 
 123  
 
 124          self.model = model 
 125          self.gensys = gen 
 126          self._autoMod = automod 
 127          self.UseAuto = False 
 128  
 
 129          if 'description' not in args: 
 130              self.description = 'None' 
 131          else: 
 132              self.description = args['description'] 
 133  
 
 134          if not hasattr(self, 'parsdict'): 
 135              self.parsdict = self.model.query('pars') 
 136          self.freepars = args['freepars'] 
 137          self.auxpars = args['auxpars'] 
 138  
 
 139          if hasattr(self, 'varslist'): 
 140              # varsindices refers to the indices in the full set of variables
 
 141              # that are used in this subset
 
 142              self.varslist.sort() 
 143              if self.curvetype == 'UD-C': 
 144                  # unused, self._systemuser -> user-supplied func will be used directly
 
 145                  self.varsindices = array([]) 
 146              else: 
 147                  orig_vars = self.model.query('vars') 
 148                  # will call self._system, selecting vars from possible ones
 
 149                  self.varsindices = array([orig_vars.index(v) for v in self.varslist]) 
 150          else: 
 151              if 'uservars' in args and self.curvetype != 'UD-C': 
 152                  self.varslist = args['uservars'] 
 153                  orig_vars = self.model.query('vars') 
 154                  # will call self._system, selecting vars from possible ones
 
 155                  self.varsindices = array([orig_vars.index(v) for v in self.varslist]) 
 156              else: 
 157                  self.varslist = self.model.query('vars') 
 158                  self.varsindices = arange(len(self.varslist)) 
 159  
 
 160          if self.gensys.haveJacobian_pars(): 
 161              fargs, fspecstr = self.gensys.funcspec._auxfnspecs['Jacobian_pars'] 
 162              Jquant = QuantSpec('J', fspecstr) 
 163              if Jquant.dim == 0: 
 164                  # dim of vars == 1 == dim of pars
 
 165                  assert len(self.varslist) == 1 
 166                  assert len(self.freepars) == 1 
 167                  # Supplied Jac w.r.t. params is custom-made for only the free params in this continuation
 
 168                  # (or there's only one parameter in system)
 
 169                  self.parsindices = array([0]) 
 170              else: 
 171                  assert len(self.varslist) == Jquant.dim 
 172                  Jquant0 = Jquant.fromvector(0) 
 173                  if Jquant0.dim == 0: 
 174                      # dim of free pars == 1
 
 175                      assert len(self.freepars) == 1 
 176                      # Supplied Jac w.r.t. params is custom-made for only the free params in this continuation
 
 177                      # (or there's only one parameter in system)
 
 178                      self.parsindices = array([0]) 
 179                  else: 
 180                      if len(self.freepars) == Jquant0.dim: 
 181                          # Supplied Jac w.r.t. params is custom-made for only the free params in this continuation
 
 182                          self.parsindices = arange(range(Jquant0.dim)) 
 183                      else: 
 184                          # Assume supplied Jac w.r.t. params is for all params in the original system
 
 185                          # therefore there should be fewer free params than # system parameters
 
 186                          assert len(self.freepars) < Jquant0.dim 
 187                          self.parsindices = array([self.parsdict.keys().index(p) for p in self.freepars]) 
 188          else: 
 189              self.parsindices = array([self.parsdict.keys().index(p) for p in self.freepars]) 
 190          self.varsdim = len(self.varslist) 
 191          self.freeparsdim = len(self.freepars) 
 192          self.auxparsdim = len(self.auxpars) 
 193          self.dim = self.varsdim + self.freeparsdim + self.auxparsdim 
 194  
 
 195          if (self.curvetype != 'UD-C'): 
 196              self.sysfunc = Function((self.dim, self.varsdim), self._system) 
 197          else: 
 198              self.sysfunc = Function((self.dim, self.varsdim), self._systemuser) 
 199  
 
 200          if (self.curvetype != 'UD-C' and self.gensys.haveJacobian()): 
 201              if self.gensys.haveJacobian_pars(): 
 202                  self.sysfunc.jac = Function((self.sysfunc.n,
 
 203                                           (self.sysfunc.m,self.sysfunc.n)),
 
 204                                          self._systemjac_withpars) 
 205              else: 
 206                  self.sysfunc.jac = Function((self.sysfunc.n,
 
 207                                           (self.sysfunc.m,self.sysfunc.n)),
 
 208                                          self._systemjac) 
 209          elif (self.curvetype == 'UD-C' and hasattr(self, '_userjac')): 
 210              self.sysfunc.jac = Function((self.sysfunc.n,
 
 211                                           (self.sysfunc.m,self.sysfunc.n)),
 
 212                                          self._systemjacuser) 
 213          else: 
 214              self.sysfunc.jac = Function((self.sysfunc.n,
 
 215                                           (self.sysfunc.m,self.sysfunc.n)),
 
 216                                          self.sysfunc.diff) 
 217  
 
 218          self.coords = self.sysfunc.coords = arange(self.varsdim).tolist() 
 219          self.params = self.sysfunc.params = (arange(self.freeparsdim \
 
 220                                                  + self.auxparsdim) \
 
 221                                                  + self.varsdim).tolist() 
 222          self.allvars = self.sysfunc.allvars = self.coords + self.params 
 223  
 
 224          # Initialize vars and pars based on initpoint
 
 225          self.initpoint = self.model.query('ics') #gensys.initialconditions.copy() 
 226          for k, v in args['initpoint'].iteritems(): 
 227              if k in self.varslist or k in args['auxpars']: 
 228                  self.initpoint[k] = v 
 229              elif k in self.model.query('pars'): 
 230                  self.parsdict[k] = v 
 231  
 
 232          for p in args['freepars']: 
 233              self.initpoint[p] = self.parsdict[p] 
 234  
 
 235          self.initpoint = tocoords(self, self.initpoint.copy()) 
 236          #self.initpoint = tocoords(self, args['initpoint'])
 
 237  
 
 238          if 'initdirec' not in args: 
 239              self.initdirec = None 
 240          else: 
 241              self.initdirec = tocoords(self, args['initdirec']) 
 242  
 
 243          if 'initcycle' not in args: 
 244              self.initcycle = None 
 245          else: 
 246              self.initcycle = args['initcycle'] 
 247  
 
 248          if not hasattr(self, "SPOut"): 
 249              self.SPOut = None 
 250          if not hasattr(self, "NumSPOut"): 
 251              self.NumSPOut = 300 
 252  
 
 253          self.preTF = None 
 254          self.reset() 
 255  
 
 256          # Removes extra parameters (first time parameter initpoint, system
 
 257          #   parameter auxpars, and uneditable parameter type) before sending
 
 258          #   to update() method
 
 259          args = dict(args) 
 260          [args.pop(i) for i in ['initpoint','initdirec','initcycle','auxpars',
 
 261                                 'type'] if i in args] 
 262          self.update(args) 
 263          self.fig = None 
 264          self.text_handles = [] 
 265          self.plot = plot 
 266  
 
 267          self._statuscodes = {0: 'Unrecognized error encountered (check stderr output).  Stopping continuation...',
 
 268                              -1: 'Do over.'}

 269  
 
 270  
 


271 -    def __copy__(self):


272          pickledself = pickle.dumps(self) 
 273          return pickle.loads(pickledself)

 274  
 


275 -    def __deepcopy__(self, memo=None, _nil=[]):


276          pickledself = pickle.dumps(self) 
 277          return pickle.loads(pickledself)

 278  
 
 279  
 


280 -    def reset(self, args=None):


281          """Resets curve by setting default parameters and deleting solution curve.""" 
 282          self.MaxNumPoints = 300 
 283          self.MaxCorrIters = 5 
 284          self.MaxTestIters = 10 
 285          self.MaxStepSize = 0.1 
 286          self.MinStepSize = 1e-5 
 287          self.StepSize = 0.01 
 288          self.VarTol = self.FuncTol = 1e-6 
 289          self.TestTol = 1e-4 
 290          self.ClosedCurve = 50 
 291          self.verbosity = 1 
 292          self.SPOut = None 
 293          self.NumSPOut = 300 
 294          self.sol = None 
 295          # record of newly computed solution segment by
 
 296          # forward or backward methods
 
 297          self.new_sol_segment = None 
 298          self.LocBifPoints = [] 
 299          self.StopAtPoints = [] 
 300          self.TestFuncs = None 
 301          self.BifPoints = {} 
 302          self.CurveInfo = PointInfo() 
 303          self.SaveJacobian = False 
 304          self.SaveEigen = False 
 305          self.Corrector = self._MoorePenrose 
 306  
 
 307          if args is not None: 
 308              self.update(args)

 309  
 
 310  
 


311 -    def update(self, args):


312          """Update parameters for Continuation.""" 
 313          if args is not None: 
 314              for k, v in args.iteritems(): 
 315                  if k in cont_args_list: 
 316                      if k == 'LocBifPoints': 
 317                          if isinstance(v, str): 
 318                              if v.lower() == 'all': 
 319                                  v = cont_bif_points 
 320                              else: 
 321                                  v = [v] 
 322  
 
 323                          # Handle stopping points
 
 324                          w = [] 
 325                          if args.has_key('StopAtPoints'): 
 326                              w = args['StopAtPoints'] 
 327                              if isinstance(w, str): 
 328                                  if w.lower() == 'all': 
 329                                      w = cont_bif_points 
 330                                  else: 
 331                                      w = [w] 
 332  
 
 333                          self.LocBifPoints = [bftype for bftype in v \
 
 334                                               if bftype in cont_bif_points] 
 335                          self.StopAtPoints = [bftype for bftype in w \
 
 336                                               if bftype in cont_bif_points] 
 337                      elif k == 'Corrector': 
 338                          self.Corrector = getattr(self, '_' + v) 
 339                      elif k != 'StopAtPoints': 
 340                          exec('self.' + k + ' = ' + repr(v)) 
 341                  elif k not in all_args_list: 
 342                      print "Warning: " + k + " is either not a valid parameter or immutable."

 343  
 
 344  
 


345 -    def _preTestFunc(self, X, V):


346          J = self.sysfunc.jac(X) 
 347          self.sysfunc.J_coords = J[:,self.coords[0]:(self.coords[-1]+1)] 
 348          self.sysfunc.J_params = J[:,self.params[0]:(self.params[-1]+1)] 
 349  
 
 350          if self.preTF is not None: 
 351              self.preTF(X, V)

 352  
 
 353  
 


354 -    def _createTestFuncs(self):


355          """Creates processors and test functions for Continuation class.
 
 356  
 
 357          Note:  In the following list, processors are in PyCont.Bifpoint
 
 358          and test functions are in PyCont.TestFunc.
 
 359  
 
 360          Point type (Processor): Test Function(s)
 
 361          ----------------------------------------
 
 362  
 
 363          BP (BranchPoint): Branch_Det
 
 364          """ 
 365          self.TestFuncs = [] 
 366          self.BifPoints = {} 
 367  
 
 368          for bftype in self.LocBifPoints: 
 369              if bftype in cont_bif_points: 
 370                  stop = bftype in self.StopAtPoints  # Set stopping flag 
 371                  if bftype is 'BP': 
 372                      method = Branch_Det(self.CorrFunc, self, save=True,
 
 373                                          numpoints=self.MaxNumPoints+1) 
 374                      self.TestFuncs.append(method) 
 375  
 
 376                      self.BifPoints['BP'] = BranchPoint(method, iszero,
 
 377                                                         stop=stop) 
 378                  elif bftype is 'B': 
 379                      method = B_Check(self.CorrFunc, self, save=True,
 
 380                                       numpoints=self.MaxNumPoints+1) 
 381                      self.TestFuncs.append(method) 
 382  
 
 383                      self.BifPoints['B'] = BPoint(method, iszero, stop=stop) 
 384  
 
 385          if self.SPOut is not None: 
 386              # add simple "user"-defined function to catch parameter values
 
 387              # during continuation
 
 388              for par, par_vals in self.SPOut.items(): 
 389                  try: 
 390                      par_ix = self.params[self.freepars.index(par)] 
 391                  except IndexError: 
 392                      raise ValueError("Invalid free parameter %s" % par) 
 393                  for i, pval in enumerate(par_vals): 
 394                      method = ParTestFunc(self.sysfunc.n,
 
 395                                           self, par_ix, pval, save=True,
 
 396                                           numpoints=self.NumSPOut+1) 
 397                      self.TestFuncs.append(method) 
 398                      self.BifPoints['SP-%s-%i' % (par, i)] = \
 
 399                          SPoint(method, iszero, stop=False)

 400  
 
 401  
 


402 -    def _system(self, X):


403          VARS = dict(zip(self.varslist, array(X)[self.coords])) 
 404          for i, par in enumerate(self.freepars): 
 405              self.parsdict[par] = X[self.params[i]] 
 406          try: 
 407              t = self.parsdict['time'] 
 408          except KeyError: 
 409              # autonomous system, t doesn't matter
 
 410              t = 0 
 411          return self.gensys.Rhs(t, VARS, self.parsdict, asarray=True)[self.varsindices]

 412  
 
 413  
 


414 -    def _systemjac(self, x0, ind=None):


415          VARS = dict(zip(self.varslist, array(x0)[self.coords])) 
 416          for i, par in enumerate(self.freepars): 
 417              self.parsdict[par] = x0[self.params[i]] 
 418          try: 
 419              t = self.parsdict['time'] 
 420          except KeyError: 
 421              # autonomous system, t doesn't matter
 
 422              t = 0 
 423          jacx = self.gensys.Jacobian(t, VARS, self.parsdict, asarray=True)[self.varsindices] 
 424          jacp = self.sysfunc.diff(x0, ind=self.params) 
 425          try: 
 426              return c_[jacx, jacp][:,ind[0]:(ind[-1]+1)] 
 427          except: 
 428              return c_[jacx, jacp]

 429  
 
 430  
 


431 -    def _systemjac_withpars(self, x0, ind=None):


432          VARS = dict(zip(self.varslist, array(x0)[self.coords])) 
 433          for i, par in enumerate(self.freepars): 
 434              self.parsdict[par] = x0[self.params[i]] 
 435          try: 
 436              t = self.parsdict['time'] 
 437          except KeyError: 
 438              # autonomous system, t doesn't matter
 
 439              t = 0 
 440          jacx = self.gensys.Jacobian(t, VARS, self.parsdict, asarray=True)[self.varsindices] 
 441          jacp = self.gensys.JacobianP(t, VARS, self.parsdict, asarray=True)[self.parsindices] 
 442          try: 
 443              return c_[jacx, jacp][:,ind[0]:(ind[-1]+1)] 
 444          except: 
 445              return c_[jacx, jacp]

 446  
 
 447  
 


448 -    def _systemuser(self, X):


449          """Calls self._userfunc, which is assumed to return an array of RHS
 
 450          values for the relevant (possibly subset of) variables.""" 
 451          VARS = dict(zip(self.varslist, array(X)[self.coords])) 
 452          for i, par in enumerate(self.freepars): 
 453              self.parsdict[par] = X[self.params[i]] 
 454          return self._userfunc(self, VARS, self.parsdict)

 455  
 
 456  
 


457 -    def _systemjacuser(self, x0, ind=None):


458          """Calls self._userjac, which is assumed to return an array of
 
 459          [Jac_x, Jac_p].""" 
 460          VARS = dict(zip(self.varslist, array(X)[self.coords])) 
 461          for i, par in enumerate(self.freepars): 
 462              self.parsdict[par] = X[self.params[i]] 
 463          return self._userjac(self, VARS, self.parsdict)

 464  
 
 465  
 


466 -    def _checkForBifPoints(self):


467          # increase efficiency by preventing many self. references
 
 468          loc = self.loc 
 469          # these declarations just make references
 
 470          curve = self.curve 
 471          V = self.V 
 472          # store commonly referenced values for efficiency
 
 473          V_loc = V[loc] 
 474          curve_loc = curve[loc] 
 475          for bftype, bfinfo in self.BifPoints.iteritems(): 
 476              flag_list = [] 
 477              for i, testfunc in enumerate(bfinfo.testfuncs): 
 478                  for k in range(testfunc.m): 
 479                      flag_list.append(bfinfo.flagfuncs[i](testfunc[loc-1][k],
 
 480                                                           testfunc[loc][k])) 
 481  
 
 482              bfpoint_found = all(flag_list) 
 483              if bfpoint_found: 
 484                  # Locate bifurcation point
 
 485                  Xval, Vval = bfinfo.locate((curve[loc-1], V[loc-1]),
 
 486                                       (curve_loc, V_loc), self) 
 487                  found = bfinfo.process(Xval, Vval, self) 
 488  
 
 489                  if found: 
 490                      # Move information one more step forward
 
 491                      if not bfinfo.stop: 
 492                          curve[loc+1] = curve_loc 
 493                          V[loc+1] = V_loc 
 494                          for testfunc in self.TestFuncs: 
 495                              testfunc[loc+1] = testfunc[loc] 
 496                      else: 
 497                          startx = copy(curve_loc) 
 498                          startv = copy(V_loc) 
 499  
 
 500                      curve[loc] = Xval 
 501                      V[loc] = Vval 
 502  
 
 503                      self._savePointInfo(loc) 
 504                      self.CurveInfo[loc] = (bftype,
 
 505                                              {'data': bfinfo.found[-1],
 
 506                                               'plot': args()}) 
 507                      if not bfinfo.stop: 
 508                          self.loc += 1 
 509                          loc += 1  # update in sync with self.loc 
 510                          V_loc = V[loc] 
 511                          curve_loc = curve[loc] 
 512                      else: 
 513                          self.CurveInfo[loc] = ('P',
 
 514                                  {'data': args(V = todict(self, startv)),
 
 515                                   'startx': todict(self, startx),
 
 516                                   'plot': args()}) 
 517                          return True 
 518  
 
 519          # Do not stop computations
 
 520          return False

 521  
 
 522  
 


523 -    def exportGeomview(self, coords=None, filename="geom.dat"):


524          if  coords is not None and len(coords) == 3: 
 525              GeomviewOutput = "(progn (geometry " + self.model.name + \
 
 526                               " { LIST {: axes_" + self.model.name + "}" 
 527              #        for cname, curve in self.curves.iteritems():
 
 528              GeomviewOutput += " {: " + self.name + "}" 
 529              GeomviewOutput += "}))\n\n" 
 530  
 
 531              # Get axes limits
 
 532              alim = [[Inf,-Inf],[Inf,-Inf],[Inf,-Inf]] 
 533              #  for cname, curve in self.curves.iteritems():
 
 534              for n in range(len(coords)): 
 535                  alim[n][0] = min(alim[n][0], min(self.sol[coords[n]])) 
 536                  alim[n][1] = max(alim[n][1], max(self.sol[coords[n]])) 
 537  
 
 538              GeomviewOutput += "(progn (hdefine geometry axes_" + \
 
 539                    self.model.name + " { appearance { linewidth 2 } SKEL 4 3 " +\
 
 540                    "0 0 0 1 0 0 0 1 0 0 0 1 " + \
 
 541                    "2 0 1 1 0 0 1 2 0 2 0 1 0 1 2 0 3 0 0 1 1})\n\n" 
 542  
 
 543              #for cname, curve in self.curves.iteritems():
 
 544              cname = self.name 
 545              GeomviewOutput += "(hdefine geometry " + cname + \
 
 546                  " { LIST {: curve_" + cname + "} {: specpts_" + cname + "}})\n\n" 
 547  
 
 548              GeomviewOutput += "(hdefine geometry curve_" + cname + \
 
 549                                " { appearance { linewidth 2 } SKEL " + \
 
 550                                repr(len(self.sol)) + " " + repr(len(self.sol)-1) 
 551              for n in range(len(self.sol)): 
 552                  GeomviewOutput += " " + repr((self.sol[n][coords[0]]-alim[0][0])/(alim[0][1]-alim[0][0])) + \
 
 553                                    " " + repr((self.sol[n][coords[1]]-alim[1][0])/(alim[1][1]-alim[1][0])) + \
 
 554                                    " " + repr((self.sol[n][coords[2]]-alim[2][0])/(alim[2][1]-alim[2][0])) 
 555              for n in range(len(self.sol)-1): 
 556                  GeomviewOutput += " 2 " + repr(n) + " " + repr(n+1) + " 0 0 0 1" 
 557  
 
 558              GeomviewOutput += "})\n\n" 
 559  
 
 560              GeomviewOutput += ")\n" 
 561  
 
 562              f = open(filename, "w") 
 563              f.write(GeomviewOutput) 
 564              f.close() 
 565          else: 
 566              raise PyDSTool_ValueError("Coordinates not specified or not of correct dimension.")

 567  
 
 568  
 


569 -    def display(self, coords=None, dirs=None, origin=None, figure=None,
 
 570                  axes=None, stability=False, domain=False, init_display=True,
 
 571                  points=True, **plot_args):


572          """Plot curve in coordinates specified by coords.
 
 573  
 
 574             Inputs:
 
 575  
 
 576                 coords -- tuple of coordinates (None defaults to the first free
 
 577                     parameter and the first state variable)
 
 578                 dirs -- tuple of coordinate directions IF coord is not in regular coords
 
 579                 origin -- Useful if want affine coordinates
 
 580          """ 
 581          # Take care of calling with state variable w/o max/min for LC
 
 582          disp_args = copy(plot_args) 
 583          if self.sol is not None: 
 584              if coords is None: 
 585                  coords = [self.freepars[0], self.varslist[0]] 
 586              if self.curvetype == 'LC-C': 
 587                  coords = list(coords) 
 588                  for n in range(2): 
 589                      if coords[n] in self.varslist: 
 590                          # Default to max of solution
 
 591                          coords[n] = coords[n]+'_max' 
 592  
 
 593              if len(coords) == 3: 
 594                  self.exportGeomview(coords=coords) 
 595                  return 
 596  
 
 597              if origin is not None: 
 598                  clist = self.sol.coordnames 
 599                  clen = len(clist) 
 600                  aorigin = array([origin[k] for k in clist]) 
 601  
 
 602              X = zeros((2,len(self.sol)), float) 
 603              for n in range(2): 
 604                  if coords[n] in self.sol.coordnames: 
 605                      X[n] = self.sol[coords[n]] 
 606                      if origin is not None: 
 607                          X[n] = X[n] - origin[coords[n]] 
 608                  elif coords[n] in self.parsdict.keys(): 
 609                      X[n] = array([self.parsdict[coords[n]]]*len(self.sol)) 
 610                      if origin is not None: 
 611                          X[n] = X[n] - origin[coords[n]] 
 612                  elif dirs is not None and coords[n] in dirs.keys(): 
 613                      # Project curve onto plane spanned by coordinate directions
 
 614                      #   spanning variables and free parameters
 
 615                      X[n] = array([matrixmultiply(x-aorigin, dirs[coords[n]]) \
 
 616                                    for x in self.sol]) 
 617                  else: 
 618                      raise KeyError('Coordinate ' + coords[n] + ' is not defined.') 
 619  
 
 620              if init_display: 
 621                  initializeDisplay(self.plot, figure=figure, axes=axes) 
 622              cfl = self.plot._cfl 
 623              cal = self.plot._cal 
 624  
 
 625              ## Prints curve
 
 626  
 
 627              # Get unique name
 
 628              name = self.name 
 629              if name in self.plot[cfl][cal]: 
 630                  num = 0 
 631                  for k, v in self.plot[cfl][cal].iteritems(): 
 632                      if isinstance(v, pargs) and k.split('_')[0] == name: 
 633                          num += 1 
 634                  name = name + '_' + repr(num) 
 635  
 
 636              self.plot[cfl][cal][name] = pargs() 
 637              self.plot[cfl][cal][name].curve = [] 
 638              label = self.curvetype.split('-')[0] 
 639              self.plot[cfl][cal][name].type = label 
 640              if stability and self.SaveEigen: 
 641                  if 'linewidth' not in disp_args:    # Default linewidth 1 
 642                      disp_args['linewidth'] = 1 
 643                  disp_args['label'] = '_nolegend_' 
 644                  stabdict = partition([x.labels[label]['stab'] \
 
 645                                        for x in self.sol],['S','U','N']) 
 646                  for stabtype, stablist in stabdict.iteritems(): 
 647                      for curve in stablist: 
 648                          self.plot[cfl][cal][name].curve.extend(pylab.plot(X[0][curve[0]:curve[1]], \
 
 649                                                                            X[1][curve[0]:curve[1]], \
 
 650                              bif_curve_colors[self.curvetype]+stab_line_styles[stabtype], **disp_args)) 
 651              else: 
 652                  if 'label' not in disp_args: 
 653                      disp_args['label'] = name 
 654                  self.plot[cfl][cal][name].curve.extend(pylab.plot(X[0], X[1], \
 
 655                                  bif_curve_colors[self.curvetype], **disp_args)) 
 656  
 
 657              # Take care of labels
 
 658              xlab = coords[0] 
 659              ylab = coords[1] 
 660              if self.curvetype == 'LC-C': 
 661                  for smtype in self.SolutionMeasures: 
 662                      if xlab.rfind('_'+smtype) > 0: 
 663                          xlab = xlab[0:xlab.rfind('_'+smtype)] 
 664                          break 
 665  
 
 666                  for smtype in self.SolutionMeasures: 
 667                      if ylab.rfind('_'+smtype) > 0: 
 668                          ylab = ylab[0:ylab.rfind('_'+smtype)] 
 669                          break 
 670  
 
 671              pylab.xlabel(xlab) 
 672              pylab.ylabel(ylab) 
 673  
 
 674              # Prints special points
 
 675              if points: 
 676                  for bftype in all_point_types: 
 677                      bflist = self.sol.bylabel(bftype) 
 678                      if bflist is not None: 
 679                          for point in bflist: 
 680                              if point.labels[bftype].has_key('name'): 
 681                                  X = zeros(2, float) 
 682                                  for n in range(2): 
 683                                      if coords[n] in self.sol.coordnames: 
 684                                          X[n] = point[coords[n]] 
 685                                          if origin is not None: 
 686                                              X[n] = X[n] - origin[coords[n]] 
 687                                      elif coords[n] in self.parsdict.keys(): 
 688                                          X[n] = self.parsdict[coords[n]] 
 689                                          if origin is not None: 
 690                                              X[n] = X[n] - origin[coords[n]] 
 691                                      elif dirs is not None and coords[n] in dirs.keys(): 
 692                                          # Project point onto plane spanned by coordinate directions
 
 693                                          #   spanning variables and free parameters
 
 694                                          X[n] = matrixmultiply(point-aorigin,
 
 695                                                                dirs[coords[n]]) 
 696  
 
 697                                  # Print point
 
 698                                  ptname = point.labels[bftype]['name'] 
 699                                  self.plot[cfl][cal][name][ptname] = pargs() 
 700                                  self.plot[cfl][cal][name][ptname].point = \
 
 701                                          pylab.plot([X[0]], [X[1]],
 
 702                                                     bif_point_colors[bftype],
 
 703                                                     label='_nolegend_') 
 704  
 
 705                                  # Print label
 
 706                                  ha = 'left' 
 707                                  if self.curvetype in ['LP-C','H-C1','H-C2','LC-C']: 
 708                                      va = 'top' 
 709                                  else: 
 710                                      va = 'bottom' 
 711  
 
 712                                  self.plot[cfl][cal][name][ptname].text = \
 
 713                                      pylab.text(X[0], X[1], '  '+ ptname,
 
 714                                                 ha=ha, va=va)

 715  
 
 716  
 


717 -    def _savePointInfo(self, loc):


718          """Created a function for this since it needs to be called
 
 719          both in _compute and when a bifurcation point is found.  It
 
 720          will have conditional statements for saving of Jacobian and
 
 721          eigenvalues, as well as other possible tidbits of
 
 722          information.""" 
 723          ptlabel = self._ptlabel 
 724          self.CurveInfo[loc] = (ptlabel, \
 
 725              {'data': args(V = todict(self, self.V[loc]),
 
 726                            ds = self.StepSize)}) 
 727  
 
 728          # Save domain information
 
 729          if 'B' in self.LocBifPoints: 
 730              val = self.BifPoints['B'].testfuncs[0][loc][0] 
 731              # if val >= 0 set domain = 'inside' otherwise 'outside'
 
 732              self.CurveInfo[loc][ptlabel]['domain'] = (val >= 0) \
 
 733                                              and 'inside' or 'outside' 
 734  
 
 735          # Save eigenvalue information
 
 736          if self.SaveEigen: 
 737              # May be able to use J_coords here
 
 738              jac = self.sysfunc.jac(self.curve[loc]) 
 739              jacx = jac[:,self.coords[0]:(self.coords[-1]+1)] 
 740              jacp = jac[:,self.params[0]:(self.params[-1]+1)] 
 741              w, vr = linalg.eig(jacx) 
 742              self.CurveInfo[loc][ptlabel]['data'].evals = w 
 743              self.CurveInfo[loc][ptlabel]['data'].evecs = vr 
 744  
 
 745              realpos = [real(eig) > 1e-6 for eig in w] 
 746              realneg = [real(eig) < -1e-6 for eig in w] 
 747              if all(realneg): 
 748                  self.CurveInfo[loc][ptlabel]['stab'] = 'S' 
 749              elif all(realpos): 
 750                  self.CurveInfo[loc][ptlabel]['stab'] = 'U' 
 751              else: 
 752                  self.CurveInfo[loc][ptlabel]['stab'] = 'N' 
 753  
 
 754          # Save jacobian information
 
 755          if self.SaveJacobian: 
 756              try: 
 757                  self.CurveInfo[loc][ptlabel]['data'].jacx = jacx 
 758                  self.CurveInfo[loc][ptlabel]['data'].jacp = jacp 
 759              except: 
 760                  jac = self.sysfunc.jac(self.curve[loc]) 
 761                  jacx = jac[:,self.coords[0]:(self.coords[-1]+1)] 
 762                  jacp = jac[:,self.params[0]:(self.params[-1]+1)] 
 763                  self.CurveInfo[loc][ptlabel]['data'].jacx = jacx 
 764                  self.CurveInfo[loc][ptlabel]['data'].jacp = jacp 
 765  
 
 766          if ptlabel == 'UD': 
 767              self.CurveInfo[loc][ptlabel]['data'].update(self._userdata)

 768  
 
 769  
 


770 -    def _MoorePenrose(self, X, V):


771          k, converged = 0, 0 
 772          problem = 0     # Only necessary for UserDefined classes 
 773          Xold = X.copy() 
 774          x0 = 0.0 
 775          x1 = 0.0 
 776          diag = args() 
 777          diag.cond = [] 
 778          diag.nrm = [] 
 779          fun = self.CorrFunc 
 780          jac = self.CorrFunc.jac 
 781          while not problem and not converged and k < self.MaxCorrIters: 
 782              A = jac(X) 
 783              B = r_[A,[V]] 
 784              R = r_[matrixmultiply(A,V),0] 
 785              Q = r_[fun(X),0] 
 786              if self.curvetype == 'UD-C' and self._userdata.has_key('problem') \
 
 787                                          and self._userdata.problem: 
 788                  problem = 1 
 789                  break 
 790              if self.verbosity >= 10: 
 791                  print "  [%d]" % k 
 792                  u, s, vh = linalg.svd(B) 
 793                  cond = s[0]/s[-1] 
 794                  diag.cond.append(cond) 
 795                  print "    Log(Condition #) = %lf" % log10(cond) 
 796              WX = linalg.solve(B,mat([R,Q]).T) 
 797              subtract(V, WX[:,0], V) 
 798              divide(V, linalg.norm(V), V) 
 799              subtract(X, WX[:,1], X) 
 800  
 
 801              # Check for convergence
 
 802              Fnrm = linalg.norm(self.CorrFunc(X)) 
 803              Vnrm = linalg.norm(WX[:,1]) 
 804              converged = Fnrm < self.FuncTol and Vnrm < self.VarTol 
 805              if self.verbosity >= 10: 
 806                  print '    (Fnrm, Vnrm) = (%.12f,%.12f)' % (Fnrm, Vnrm) 
 807                  x0 = x1 
 808                  x1 = linalg.norm(X-Xold) 
 809                  Xold = X.copy() 
 810                  diag.nrm.append((Fnrm, Vnrm)) 
 811              k += 1 
 812          return k, converged, problem, diag

 813  
 
 814  
 


815 -    def _Natural(self, X, V):


816          # Get index of coordinate with maximal change
 
 817          k, converged = 0, 0 
 818          problem = 0     # Only necessary for UserDefined classes 
 819          Xold = X.copy()    # Use for secant predictor below 
 820          x0 = 0.0 
 821          x1 = 0.0 
 822          diag = args() 
 823          diag.cond = [] 
 824          diag.nrm = [] 
 825  
 
 826          ind = argsort(V)[-1] 
 827          vi = zeros(len(X), float64) 
 828          vi[ind] = 1.0 
 829          xi = X[ind] 
 830          fun = self.CorrFunc 
 831          jac = self.CorrFunc.jac 
 832          while not problem and not converged and k < self.MaxCorrIters: 
 833              # Newton's method: X_{n+1} = X_{n} - W, where W = B^{-1}*Q
 
 834              A = jac(X) 
 835              B = r_[A,[vi]] 
 836              Q = r_[fun(X), X[ind] - xi] 
 837              if self.curvetype == 'UD-C' and self._userdata.has_key('problem') \
 
 838                                          and self._userdata.problem: 
 839                  problem = 1 
 840                  break 
 841              if self.verbosity >= 10: 
 842                  print "  [%d]" % k 
 843                  u, s, vh = linalg.svd(B) 
 844                  cond = s[0]/s[-1] 
 845                  diag.cond.append(cond) 
 846                  print "    Log(Condition #) = %lf" % log10(cond) 
 847              W = linalg.solve(B, Q) 
 848              subtract(X, W, X) 
 849  
 
 850              # Check for convergence
 
 851              Fnrm = linalg.norm(self.CorrFunc(X)) 
 852              Vnrm = linalg.norm(W) 
 853              converged = Fnrm < self.FuncTol and Vnrm < self.VarTol 
 854              if self.verbosity >= 10: 
 855                  print '    (Fnrm, Vnrm) = (%.12f,%.12f)' % (Fnrm, Vnrm) 
 856                  x0 = x1 
 857                  x1 = linalg.norm(X-Xold) 
 858                  Xold = X.copy() 
 859                  diag.nrm.append((Fnrm, Vnrm)) 
 860              k += 1 
 861  
 
 862          V = linalg.solve(r_[A,[V]], r_[zeros((self.varsdim, 1), float), [[1.]]]) 
 863          V = V/linalg.norm(V) 
 864          return k, converged, problem, diag

 865  
 
 866  
 


867 -    def _Keller(self, X, V):


868          k, converged = 0, 0 
 869          problem = 0     # Only necessary for UserDefined classes 
 870          Xold = X.copy() 
 871          x0 = 0.0 
 872          x1 = 0.0 
 873          diag = args() 
 874          diag.cond = [] 
 875          diag.nrm = [] 
 876          fun = self.CorrFunc 
 877          jac = self.CorrFunc.jac 
 878          while not problem and not converged and k < self.MaxCorrIters: 
 879              A = jac(X) 
 880              B = r_[A,[V]] 
 881              Q = r_[fun(X),
 
 882                     matrixmultiply(X-self.curve[self.loc-1],V)-self.StepSize] 
 883              if self.curvetype == 'UD-C' and self._userdata.has_key('problem') \
 
 884                 and self._userdata.problem: 
 885                  problem = 1 
 886                  break 
 887              if self.verbosity >= 10: 
 888                  print "  [%d]" % k 
 889                  u, s, vh = linalg.svd(B) 
 890                  cond = s[0]/s[-1] 
 891                  diag.cond.append(cond) 
 892                  print "    Log(Condition #) = %lf" % log10(cond) 
 893              W = linalg.solve(B, Q) 
 894              subtract(X, W, X) 
 895  
 
 896              # Check for convergence
 
 897              Fnrm = linalg.norm(self.CorrFunc(X)) 
 898              Vnrm = linalg.norm(W) 
 899              converged = Fnrm < self.FuncTol and Vnrm < self.VarTol 
 900              if self.verbosity >= 10: 
 901                  print '    (Fnrm, Vnrm) = (%.12f,%.12f)' % (Fnrm, Vnrm) 
 902                  x0 = x1 
 903                  x1 = linalg.norm(X-Xold) 
 904                  Xold = X.copy() 
 905                  diag.nrm.append((Fnrm, Vnrm)) 
 906              k += 1 
 907  
 
 908          V = linalg.solve(r_[A,[V]], r_[zeros((self.varsdim,1), float), [[1.]]]) 
 909          V = V/linalg.norm(V) 
 910          return k, converged, problem, diag

 911  
 
 912  
 


913 -    def _compute(self, x0=None, v0=None, direc=1):


914          """Continuation using Moore-Penrose method (called by forward
 
 915          and backward methods)
 
 916  
 
 917          NOTE: For codimension 2 curves, CorrFunc is the augmented
 
 918          system consisting of sysfunc with testfunc associated with the
 
 919          curve given by sysfunc.  When you call CorrFunc, it calls
 
 920          sysfunc.PreTestFunc to calculate the jacobian that testfunc
 
 921          needs.  THUS, sysfunc jacobians are computed and stored in
 
 922          sysfunc.  Now, if you have test functions that require
 
 923          jacobian information from sysfunc and CorrFunc, I only call
 
 924          CorrFunc.PreTestFunc. BUT, it still works because CorrFunc was
 
 925          called just previous, thereby saving sysfunc jacobians that
 
 926          are needed by the test functions that rely on sysfunc
 
 927          jacobians.  Understand? Good, cuz I barely do.  I'm going to
 
 928          try and alleviate this confusion.  Wish me luck...  By the
 
 929          way, the example of this is in computing ZH points.  They
 
 930          require test functions for a codimension 1 curve while
 
 931          continuing a codimension 2 curve.
 
 932          """ 
 933  
 
 934          # Process X
 
 935          if x0 is None: 
 936              x0 = self.initpoint 
 937  
 
 938          if isinstance(x0, dict): 
 939              x0 = tocoords(self, x0) 
 940          elif isinstance(x0, list): 
 941              x0 = array(x0) 
 942          elif isinstance(x0, Point): 
 943              x0 = x0.todict() 
 944              for p in self.freepars: 
 945                  if p not in x0.keys(): 
 946                      x0[p] = self.parsdict[p] 
 947              x0 = tocoords(self, x0) 
 948  
 
 949          # Get Jacobian information
 
 950          if self.sysfunc == self.CorrFunc: 
 951              # It's codim 1 and can use the existing Jac (in case it's symbolic)
 
 952              J = self.CorrFunc.jac(x0) 
 953              J_coords = J[:,self.coords] 
 954              J_params = J[:,self.params] 
 955          else: 
 956              # SHORTCOMING HERE:
 
 957              # replace Jac with numerical diff, regardless of whether
 
 958              # original sysfunc had a symbolic Jac
 
 959              self.CorrFunc.jac = Function((self.CorrFunc.n,
 
 960                                        (self.CorrFunc.m,self.CorrFunc.n)), \
 
 961                            self.CorrFunc.diff, numpoints=self.MaxNumPoints+1) 
 962              J_coords = self.CorrFunc.jac(x0, self.coords) 
 963              J_params = self.CorrFunc.jac(x0, self.params) 
 964  
 
 965          # Process V
 
 966          if v0 is None: 
 967              # Compute tangent vector v0 to curve at ix0 by solving:
 
 968              #   [A; rand(n)] * v0 = [zeros(n-1); 1]
 
 969              #
 
 970              v0 = zeros(self.dim, float) 
 971              v0 = linalg.solve(r_[c_[J_coords, J_params],
 
 972                                   [2*(random(self.dim)-0.5)]], \
 
 973                                r_[zeros(self.dim-1),1]) 
 974              v0 /= linalg.norm(v0) 
 975              v0 = direc*sign([x for x in v0 if abs(x) > 1e-8][0])*v0 
 976          elif isinstance(v0, dict): 
 977              v0 = direc*tocoords(self, v0) 
 978          elif isinstance(v0, list): 
 979              v0 = direc*array(v0) 
 980          elif isinstance(v0, Point): 
 981              v0 = direc*v0.toarray() 
 982  
 
 983          self.V = zeros((self.MaxNumPoints+1,self.dim), float) 
 984          self.V[0] = v0 
 985  
 
 986          # Start on curve
 
 987          # NOTE: If having trouble with losing branch given by initdirec, then save
 
 988          #   self.V[0] = v0.copy() and replace after.
 
 989          self.curve = zeros((self.MaxNumPoints+1, self.dim), float) 
 990          self.curve[0] = x0 
 991  
 
 992          # curve and V are arrays and so will be copied by reference only
 
 993          curve = self.curve 
 994          V = self.V 
 995  
 
 996          converged = True 
 997          attempts = 0 
 998          val = linalg.norm(self.CorrFunc(x0)) 
 999          while val > self.FuncTol or not converged: 
1000              try: 
1001                  k, converged, problem, diag = self.Corrector(curve[0], V[0]) 
1002              except: 
1003                  converged = False 
1004                  print "Error occurred in dynamical system computation" 
1005              else: 
1006                  val = linalg.norm(self.CorrFunc(curve[0])) 
1007              attempts += 1 
1008              if not converged and attempts > 1: 
1009                  # Stop continuation
 
1010                  raise PyDSTool_ExistError("Could not find starting point on curve.  Stopping continuation.") 
1011          # Initialize index location on curve data set
 
1012          self.loc = 0 
1013          if self.verbosity >= 3: 
1014              print '    Found initial point on curve.' 
1015  
 
1016          # Initialize test functions
 
1017          self._createTestFuncs() 
1018  
 
1019          x0 = curve[0] 
1020          v0 = V[0] 
1021          J = self.CorrFunc.jac(x0) 
1022          self.CorrFunc.J_coords = J[:,self.coords] 
1023          self.CorrFunc.J_params = J[:,self.params] 
1024  
 
1025          if self.TestFuncs != []: 
1026              for testfunc in self.TestFuncs: 
1027                  if hasattr(testfunc, 'setdata'): 
1028                      testfunc.setdata(x0, v0) 
1029              self._preTestFunc(x0, v0) 
1030              for testfunc in self.TestFuncs: 
1031                  testfunc[self.loc] = testfunc(x0, v0) 
1032  
 
1033          # Save initial information
 
1034          self._savePointInfo(self.loc) 
1035          self.CurveInfo[0] = ('P', {'data': args(V = todict(self, v0)), \
 
1036                                     'plot': args()}) 
1037  
 
1038          # Stepsize control parameters
 
1039          CorrThreshold = 6 
1040          SSC_c, SSC_C = 0.8, 1.2 
1041  
 
1042          if self.verbosity >= 3: 
1043              print '    Beginning continuation...' 
1044          # Continuation loop
 
1045          closed = False 
1046          stop = False 
1047          problem = False 
1048          if self.curvetype == 'UD-C' and self._userdata.has_key('problem'): 
1049              self._userdata.problem = False 
1050  
 
1051          # de-references to improve efficiency
 
1052          loc = self.loc  # integer, so self.loc won't get updated automatically 
1053          while loc+1 < self.MaxNumPoints and not stop: 
1054              # Predictor
 
1055              loc += 1 
1056              curve[loc] = curve[loc-1] + self.StepSize*V[loc-1] 
1057              V[loc] = V[loc-1] 
1058  
 
1059              # Corrector -- update self.loc for Corrector's reference
 
1060              self.loc = loc 
1061              try: 
1062                  k, converged, problem, diag = self.Corrector(curve[loc], V[loc]) 
1063              except: 
1064                  problem = True 
1065              #if self._userdata.has_key('problem'):  # Uncomment the these three lines to "find" the boundary between regions
 
1066              #    self._userdata.problem = False
 
1067              #    problem = False
 
1068              if self.verbosity >= 10: 
1069                  print "Step #%d:" % loc 
1070                  print "  Corrector steps: %d/%d" % (k, self.MaxCorrIters) 
1071                  print "  Converged: %d" % (converged and not problem) 
1072  
 
1073              if problem: 
1074                  stop = True 
1075              elif not converged: 
1076                  loc -= 1 
1077                  if self.StepSize > self.MinStepSize: 
1078                      # Reduce stepsize and try again
 
1079                      if self.verbosity >= 3: 
1080                          print "Trouble converging.  Reducing stepsize. (ds=%lf)" % self.StepSize 
1081                      self.StepSize = max(self.MinStepSize, self.StepSize*SSC_c) 
1082                  else: 
1083                      # Stop continuation
 
1084                      print "Did not converge.  Stopping continuation.  Reduce MinStepSize to continue." 
1085                      raise PyDSTool_ExistError("Did not converge. Stopping continuation. Reduce MinStepSize to continue") 
1086              else: 
1087                  # Increase stepsize for fast convergence
 
1088                  if self.StepSize < self.MaxStepSize and k < CorrThreshold: 
1089                      self.StepSize = min(self.MaxStepSize, self.StepSize*SSC_C) 
1090  
 
1091                  # Evaluate test functions
 
1092                  if self.TestFuncs is not None: 
1093                      self._preTestFunc(curve[loc], V[loc]) 
1094                      for testfunc in self.TestFuncs: 
1095                          testfunc[loc] = testfunc(curve[loc], V[loc]) 
1096  
 
1097                  # Check for bifurcation points.
 
1098                  # If _checkForBifPoints returns True, stop loop
 
1099                  # update self.loc for Corrector's reference
 
1100                  self.loc = loc 
1101                  if self.BifPoints != {} and self._checkForBifPoints(): 
1102                      stop = True 
1103  
 
1104                  # Checks to see if curve is closed and if closed, it closes the curve
 
1105                  if self.ClosedCurve < loc+1 < self.MaxNumPoints and \
 
1106                  linalg.norm(curve[loc]-curve[0]) < self.StepSize: 
1107                      # Need to be able to copy PointInfo information
 
1108                      curve[loc+1] = curve[0] 
1109                      V[loc+1] = V[0] 
1110  
 
1111                      for testfunc in self.TestFuncs: 
1112                          testfunc[loc+1] = testfunc[loc] 
1113  
 
1114                      self._savePointInfo(loc) 
1115                      loc += 1 
1116                      self._savePointInfo(loc) 
1117  
 
1118                      closed = True 
1119                      break 
1120  
 
1121                  # Print information
 
1122                  if self.verbosity >= 4: 
1123                      print "Loc = %4d    %s = %lf" % (loc, self.freepars[0],
 
1124                                                    curve[loc][-1]) 
1125  
 
1126                  # Save information
 
1127                  self._savePointInfo(loc) 
1128  
 
1129          # Finish updating self.loc integer location
 
1130          self.loc = loc 
1131          # Save end point information
 
1132          if problem: 
1133              #self.CurveInfo[loc] = ('UD', {'data': args(V = todict(self,
 
1134              #                            V[self.loc]), ds=self.StepSize)})
 
1135              self.CurveInfo[loc] = ('MX', {'data': args(V = todict(self,
 
1136                                          V[loc]), ds=self.StepSize)}) 
1137          elif not closed and not self.CurveInfo[loc].has_key('P'): 
1138              self.CurveInfo[loc] = ('P', {'data': args(V = todict(self,
 
1139                                          V[loc])), 'plot': args()})

1140  
 
1141  
 


1142 -    def forward(self):


1143          """Computes forward along curve from initpoint if this is the first run.  Otherwise, it computes
 
1144          forward along curve from the last point on the saved solution sol.  The new curve is appended to
 
1145          the end of sol.""" 
1146          #if self.gensys.haveJacobian_pars():
 
1147          #    raise NotImplementedError("Jacobian with respect to parameters is not currently implemented in PyCont.")
 
1148  
 
1149          self.CurveInfo = PointInfo() 
1150          if self.sol is None: 
1151              self._compute(v0=self.initdirec) 
1152              self.sol = self._curveToPointset() 
1153  
 
1154              for pttype in self.LocBifPoints + other_special_points: 
1155                  bylabels = self.sol.bylabel(pttype) 
1156                  if bylabels is not None: 
1157                      num = 1 
1158                      for pt in bylabels: 
1159                          if pttype in pt.labels: 
1160                              pt.labels[pttype]['name'] = pttype + repr(num) 
1161                          else: 
1162                              pt.labels[pttype] = {'name': pttype + repr(num)} 
1163                          if pt.labels[pttype].has_key('cycle'): 
1164                              pt.labels[pttype]['cycle'].name = pttype + repr(num) 
1165                          num += 1 
1166              self.new_sol_segment = copy(self.sol) 
1167          else: 
1168              sol1 = self.sol[-1] 
1169              # Set start point (if bif point, set to startx)
 
1170              if sol1.labels['P'].has_key('startx'): 
1171                  x0 = sol1.labels['P']['startx'] 
1172              else: 
1173                  x0 = sol1 
1174  
 
1175              try: 
1176                  v0 = sol1.labels['P']['data'].V 
1177              except: 
1178                  try: 
1179                      v0 = sol1.labels['MX']['data'].V 
1180                  except: 
1181                      v0 = None 
1182  
 
1183              if sol1.labels[self.curvetype.split('-')[0]]['data'].has_key('ds'): 
1184                  self.StepSize = min(self.StepSize,
 
1185                            sol1.labels[self.curvetype.split('-')[0]]['data'].ds) 
1186  
 
1187              self._compute(x0=x0, v0=v0) 
1188              sol = self._curveToPointset()[1:] 
1189  
 
1190              # Fix labels
 
1191              try: 
1192                  self.sol.labels.remove(len(self.sol)-1,'P') 
1193              except: 
1194                  pass 
1195  
 
1196              try: 
1197                  self.sol.labels.remove(len(self.sol)-1,'MX') 
1198              except: 
1199                  pass 
1200  
 
1201              for pttype in self.LocBifPoints + other_special_points: 
1202                  if self.sol.bylabel(pttype) is not None: 
1203                      num = len(self.sol.bylabel(pttype)) + 1 
1204                  else: 
1205                      num = 1 
1206                  bylabels = sol.bylabel(pttype) 
1207                  if bylabels is not None: 
1208                      for pt in bylabels: 
1209                          if pttype in pt.labels: 
1210                              pt.labels[pttype]['name'] = pttype + repr(num) 
1211                          else: 
1212                              pt.labels[pttype] = {'name': pttype + repr(num)} 
1213                          if pt.labels[pttype].has_key('cycle'): 
1214                              pt.labels[pttype]['cycle'].name = pttype + repr(num) 
1215                          num += 1 
1216  
 
1217              self.new_sol_segment = copy(sol) 
1218              self.sol.append(sol)

1219  
 
1220  
 


1221 -    def backward(self):


1222          """Computes backward along curve from initpoint if this is the
 
1223          first run.  Otherwise, it computes backward along curve from
 
1224          the first point on the saved solution sol.  The new curve is
 
1225          appended to the front of sol.
 
1226          """ 
1227          #if self.gensys.haveJacobian_pars():
 
1228          #    raise NotImplementedError('Jacobian with respect to parameters is not currently implemented in PyCont.')
 
1229  
 
1230          self.CurveInfo = PointInfo() 
1231          if self.sol is None: 
1232              self._compute(v0=self.initdirec, direc=-1) 
1233              self.sol = self._curveToPointset() 
1234  
 
1235              # Turn curve around
 
1236              self.sol.reverse() 
1237              sol0 = self.sol[0] 
1238  
 
1239              # Type of end point
 
1240              etype0 = sol0.labels.has_key('P') and 'P' or 'MX' 
1241              etype1 = self.sol[-1].labels.has_key('P') and 'P' or 'MX' 
1242  
 
1243              # Turn tangent vectors around (for non auto only)
 
1244              if not self.UseAuto: 
1245                  # Turn tangent vectors at end point type around
 
1246                  for k, v in sol0.labels[etype0]['data'].V.iteritems(): 
1247                      sol0.labels[etype0]['data'].V[k] = -1*v 
1248  
 
1249                  for k, v in self.sol[-1].labels[etype1]['data'].V.iteritems(): 
1250                      self.sol[-1].labels[etype1]['data'].V[k] = -1*v 
1251  
 
1252                  # Turn tangent vectors at curve type around
 
1253                  ctype = self.curvetype.split('-')[0] 
1254                  for pt in self.sol: 
1255                      for k, v in pt.labels[ctype]['data'].V.iteritems(): 
1256                          pt.labels[ctype]['data'].V[k] = -1*v 
1257  
 
1258              for pttype in self.LocBifPoints + other_special_points: 
1259                  bylabels = self.sol.bylabel(pttype) 
1260                  if bylabels is not None: 
1261                      num = 1 
1262                      for pt in bylabels: 
1263                          if pttype in pt.labels: 
1264                              pt.labels[pttype]['name'] = pttype + repr(num) 
1265                          else: 
1266                              pt.labels[pttype] = {'name': pttype + repr(num)} 
1267                          if pt.labels[pttype].has_key('cycle'): 
1268                              pt.labels[pttype]['cycle'].name = pttype + repr(num) 
1269                          num += 1 
1270              self.new_sol_segment = copy(self.sol) 
1271          else: 
1272              sol0 = self.sol[0] 
1273              # Set start point (if bif point, set to startx)
 
1274              if sol0.labels['P'].has_key('startx'): 
1275                  x0 = sol0.labels['P']['startx'] 
1276              else: 
1277                  x0 = sol0 
1278  
 
1279              try: 
1280                  v0 = sol0.labels['P']['data'].V 
1281              except: 
1282                  try: 
1283                      v0 = sol0.labels['MX']['data'].V 
1284                  except: 
1285                      v0 = None 
1286  
 
1287              ctype = self.curvetype.split('-')[0] 
1288              #try:
 
1289              #    self.StepSize = self.sol[0].labels[ctype]['data'].ds
 
1290              #except:
 
1291              #    pass
 
1292              if sol0.labels[self.curvetype.split('-')[0]]['data'].has_key('ds'): 
1293                  self.StepSize = min(self.StepSize,
 
1294                      sol0.labels[self.curvetype.split('-')[0]]['data'].ds) 
1295  
 
1296              self._compute(x0=x0, v0=v0, direc=-1) 
1297              sol = self._curveToPointset() 
1298  
 
1299              # Turn curve around
 
1300              sol.reverse() 
1301              sol0 = sol[0] 
1302  
 
1303              # Type of end point
 
1304              etype = sol0.labels.has_key('P') and 'P' or 'MX' 
1305  
 
1306              if etype in sol0.labels: 
1307                  sol0.labels[etype]['name'] = etype+'1' 
1308              else: 
1309                  sol0.labels[etype] = {'name': etype+'1'} 
1310              if sol0.labels[etype].has_key('cycle'): 
1311                  sol0.labels[etype]['cycle'].name = etype+'1' 
1312  
 
1313              # Turn tangent vectors around (for non auto only)
 
1314              if not self.UseAuto: 
1315                  # Turn tangent vector around at point type endtype and change name
 
1316                  for k, v in sol0.labels[etype]['data'].V.iteritems(): 
1317                      sol0.labels[etype]['data'].V[k] = -1*v 
1318  
 
1319                  # Turn tangent vectors at curve type around
 
1320                  for pt in sol: 
1321                      for k, v in pt.labels[ctype]['data'].V.iteritems(): 
1322                          pt.labels[ctype]['data'].V[k] = -1*v 
1323  
 
1324              # Fix labels
 
1325              try: 
1326                  self.sol.labels[0].pop('P') 
1327              except: 
1328                  self.sol.labels[0].pop('MX') 
1329  
 
1330              for pttype in self.LocBifPoints + ['RG', 'UZ', 'MX']: 
1331                  bylabels = self.sol.bylabel(pttype) 
1332                  if bylabels is not None: 
1333                      num = len(bylabels) + 1 
1334                  else: 
1335                      num = 1 
1336                  new_bylabels = sol.bylabel(pttype) 
1337                  if new_bylabels is not None: 
1338                      for pt in new_bylabels: 
1339                          if pttype in pt.labels: 
1340                              pt.labels[pttype]['name'] = pttype + repr(num) 
1341                          else: 
1342                              pt.labels[pttype] = {'name': pttype + repr(num)} 
1343                          if pt.labels[pttype].has_key('cycle'): 
1344                              pt.labels[pttype]['cycle'].name = pttype + repr(num) 
1345                          num += 1 
1346  
 
1347              self.new_sol_segment = copy(sol) 
1348              sol.append(self.sol) 
1349              self.sol = sol

1350  
 
1351  
 


1352 -    def testdomain(self, ic=None, ind=0, direc=1):


1353          if ic is None: 
1354              ic = self.initpoint 
1355          else: 
1356              x0 = ic.copy() 
1357              x0 = x0.todict() 
1358              for p in self.freepars: 
1359                  if p not in x0.keys(): 
1360                      x0[p] = self.parsdict[p] 
1361              x0 = tocoords(self, x0) 
1362  
 
1363          dy = 0.5 
1364          Dy = 0.0 
1365          self.CorrFunc.jac = Function((self.CorrFunc.n,
 
1366                                        (self.CorrFunc.m,self.CorrFunc.n)), \
 
1367                                  self.CorrFunc.diff, numpoints=1) 
1368  
 
1369          # Process V
 
1370          try: 
1371              icv = ic.labels['UD']['data'].V 
1372          except: 
1373              icv = None 
1374  
 
1375          if icv is None: 
1376              # Get Jacobian information
 
1377              print "Creating vector...\n" 
1378              J_coords = self.CorrFunc.jac(x0, self.coords) 
1379              J_params = self.CorrFunc.jac(x0, self.params) 
1380  
 
1381              v0 = zeros(self.dim, float) 
1382              v0 = linalg.solve(r_[c_[J_coords, J_params],
 
1383                                   [2*(random(self.dim)-0.5)]], \
 
1384                                r_[zeros(self.dim-1),1]) 
1385              v0 /= linalg.norm(v0) 
1386              v0 = array(sign([x for x in v0 if abs(x) > 1e-8][0])*v0) 
1387          else: 
1388              v0 = icv.copy() 
1389              v0 = tocoords(self, v0) 
1390  
 
1391          ic = x0.copy() 
1392          icv = v0.copy() 
1393          #while (abs(x0[ind] - ic[ind]) < 1.0):
 
1394          out = [] 
1395          while (dy >= 1e-4): 
1396              print "%s = %lf" % (self.varslist[ind], x0[ind]) 
1397              # Get Jacobian information
 
1398              #J_coords = self.CorrFunc.jac(x0, self.coords)
 
1399              #J_params = self.CorrFunc.jac(x0, self.params)
 
1400  
 
1401              k, converged, problem, diag = self.Corrector(x0, v0) 
1402              print 'x0 = ', x0 
1403              if not converged: 
1404                  print "  Did not converge." 
1405                  if (Dy >= dy): 
1406                      print "  Changing stepsize." 
1407                      Dy -= dy 
1408                      dy *= 0.5 
1409                  else: 
1410                      print "  Minimum reached.  Stopping simulation." 
1411                      raise PyDSTool_ExistError("Failed to converge") 
1412              else: 
1413                  out.append((Dy, diag)) 
1414                  #print "  Converged.  Avg. cond. # = %lf" % (csum/k)
 
1415                  print "  Converged.  Avg. cond. # = %lf" % (sum(diag.cond)/len(diag.cond)) 
1416  
 
1417                  # Takes care of "infinite" domains
 
1418                  if (Dy >= 5.0): 
1419                      Dy -= dy 
1420                      dy *= 0.5 
1421  
 
1422              self._userdata.problem = False 
1423  
 
1424              x0 = ic.copy() 
1425              Dy += dy 
1426              x0[ind] += direc*Dy 
1427  
 
1428          return out

1429  
 
1430  
 


1431 -    def testdomaingrid(self, ic=None, coords=('y', 'theta'), Dx=None, Dy=None, step=2):


1432          key = ['y', 'theta', 'a'] 
1433          ind = (key.index(coords[0]), key.index(coords[1])) 
1434  
 
1435          if ic is None: 
1436              ic = self.initpoint 
1437          else: 
1438              ic = self.sol[ic] 
1439              x0 = ic.copy() 
1440              x0 = x0.todict() 
1441              for p in self.freepars: 
1442                  if p not in x0.keys(): 
1443                      x0[p] = self.parsdict[p] 
1444              x0 = tocoords(self, x0) 
1445  
 
1446          self.CorrFunc.jac = Function((self.CorrFunc.n,
 
1447                                        (self.CorrFunc.m,self.CorrFunc.n)), \
 
1448                                  self.CorrFunc.diff, numpoints=1) 
1449  
 
1450          # Process V
 
1451          try: 
1452              icv = ic.labels['UD']['data'].V 
1453          except: 
1454              icv = None 
1455  
 
1456          if icv is None: 
1457              # Get Jacobian information
 
1458              print "Creating vector...\n" 
1459              J_coords = self.CorrFunc.jac(x0, self.coords) 
1460              J_params = self.CorrFunc.jac(x0, self.params) 
1461  
 
1462              v0 = zeros(self.dim, float) 
1463              v0 = linalg.solve(r_[c_[J_coords, J_params],
 
1464                                   [2*(random(self.dim)-0.5)]], \
 
1465                                r_[zeros(self.dim-1),1]) 
1466              v0 /= linalg.norm(v0) 
1467              v0 = array(sign([x for x in v0 if abs(x) > 1e-8][0])*v0) 
1468          else: 
1469              v0 = icv.copy() 
1470              v0 = tocoords(self, v0) 
1471  
 
1472          out = [] 
1473          if Dx is None: 
1474              xmin = min([pt[coords[0]] for pt in self.sol]) 
1475              xmax = max([pt[coords[0]] for pt in self.sol]) 
1476          else: 
1477              xmin = x0[ind[0]]-Dx 
1478              xmax = x0[ind[0]]+Dx 
1479          Dx = xmax-xmin 
1480          dx = Dx/(2*step) 
1481  
 
1482          if Dy is None: 
1483              ymin = min([pt[coords[1]] for pt in self.sol]) 
1484              ymax = max([pt[coords[1]] for pt in self.sol]) 
1485          else: 
1486              ymin = x0[ind[1]]-Dy 
1487              ymax = x0[ind[1]]+Dy 
1488          Dy = ymax-ymin 
1489          dy = Dy/(2*step) 
1490  
 
1491          ic = array(x0.copy()) 
1492          icv = array(v0.copy()) 
1493          for i in range(2*step+1): 
1494              out.append([]) 
1495              for j in range(2*step+1): 
1496                  #x0 = array(ic.copy())
 
1497                  #v0 = array(icv.copy())
 
1498                  x0 = ic.copy() 
1499                  v0 = icv.copy() 
1500                  x0[ind[0]] = xmin + j*dx 
1501                  x0[ind[1]] = ymax - i*dy 
1502                  ix0 = x0.copy() 
1503                  print "(%d, %d) -- (%s, %s) = (%lf, %lf)" % (i, j, key[ind[0]], key[ind[1]], x0[ind[0]], x0[ind[1]]) 
1504  
 
1505                  # Get Jacobian information
 
1506                  #J_coords = self.CorrFunc.jac(x0, self.coords)
 
1507                  #J_params = self.CorrFunc.jac(x0, self.params)
 
1508  
 
1509                  #v0 = zeros(self.dim, float)
 
1510                  #v0 = linalg.solve(r_[c_[J_coords, J_params], [2*(random(self.dim)-0.5)]], \
 
1511                  #    r_[zeros(self.dim-1),1])
 
1512                  #v0 /= linalg.norm(v0)
 
1513                  #v0 = array(sign([x for x in v0 if abs(x) > 1e-8][0])*v0)
 
1514  
 
1515                  k, converged, problem, diag = self.Corrector(x0, v0) 
1516  
 
1517                  print 'x0 = ', x0 
1518                  if not converged: 
1519                      print "  Did not converge." 
1520                      if len(diag.cond) > 0: 
1521                          print "  Avg. cond. # = %lf" % (sum(diag.cond)/len(diag.cond)) 
1522                      if problem: 
1523                          # Did not converge due to failure in backward integration
 
1524                          out[i].append(('XB', ix0, x0, diag)) 
1525                      elif k >= self.MaxCorrIters: 
1526                          Fnm = [nm[0] for nm in diag.nrm] 
1527                          if Fnm[-1] < Fnm[-2] and Fnm[-2] < Fnm2[-3]: 
1528                              # Did not converge but may have converged with more timesteps
 
1529                              out[i].append(('XC', ix0, x0, diag)) 
1530                          else: 
1531                              # Did not converge for unknown reason
 
1532                              out[i].append(('X', ix0, x0, diag)) 
1533                  else: 
1534                      print "  Converged.  Avg. cond. # = %lf" % (sum(diag.cond)/len(diag.cond)) 
1535                      # Converged
 
1536                      out[i].append(('C', ix0, x0, diag)) 
1537  
 
1538                  self._userdata.problem = False 
1539  
 
1540          return out

1541  
 
1542  
 


1543 -    def testdomaintangrid(self, Dx, Dy, ic=None, step=2):


1544          key = ['y', 'theta', 'a'] 
1545  
 
1546          if ic is None: 
1547              ic = self.initpoint 
1548          else: 
1549              ic = self.sol[ic] 
1550              x0 = ic.copy() 
1551              x0 = x0.todict() 
1552              for p in self.freepars: 
1553                  if p not in x0.keys(): 
1554                      x0[p] = self.parsdict[p] 
1555              x0 = tocoords(self, x0) 
1556  
 
1557          self.CorrFunc.jac = Function((self.CorrFunc.n,
 
1558                                        (self.CorrFunc.m,self.CorrFunc.n)), \
 
1559                                  self.CorrFunc.diff, numpoints=1) 
1560  
 
1561          # Process V
 
1562          try: 
1563              icv = ic.labels['UD']['data'].V 
1564          except: 
1565              icv = None 
1566  
 
1567          if icv is None: 
1568              # Get Jacobian information
 
1569              print "Creating vector...\n" 
1570              J_coords = self.CorrFunc.jac(x0, self.coords) 
1571              J_params = self.CorrFunc.jac(x0, self.params) 
1572  
 
1573              v0 = zeros(self.dim, float) 
1574              v0 = linalg.solve(r_[c_[J_coords, J_params],
 
1575                                   [2*(random(self.dim)-0.5)]], \
 
1576                                r_[zeros(self.dim-1),1]) 
1577              v0 /= linalg.norm(v0) 
1578              v0 = array(sign([x for x in v0 if abs(x) > 1e-8][0])*v0) 
1579          else: 
1580              v0 = icv.copy() 
1581              v0 = tocoords(self, v0) 
1582  
 
1583          v1 = zeros(self.dim, float) 
1584          v1[2] = 1.0 
1585          v1 = v1 - v0[2]*v0 
1586          v1 = v1/linalg.norm(v1) 
1587          print "Checking orthonormal..." 
1588          print "  |v0| = %lf" % linalg.norm(v0) 
1589          print "  |v1| = %lf" % linalg.norm(v1) 
1590          print "  <v0,v1> = %lf" % matrixmultiply(v0,v1) 
1591  
 
1592          out = [] 
1593          d0 = Dx/(2*step) 
1594          d1 = Dy/(2*step) 
1595  
 
1596          print "Start x = ", x0 
1597          for i in range(step, -1*(step+1), -1): 
1598              out.append([]) 
1599              for j in range(-1*step, step+1): 
1600                  x = x0 + j*d0*v0 + i*d1*v1 
1601                  print "(%d, %d) -- (y, theta, a) = (%lf, %lf, %lf)" % (i, j, x[0], x[1], x[2]) 
1602  
 
1603                  ix = x.copy() 
1604                  v = v0.copy() 
1605  
 
1606                  k, converged, problem, diag = self.Corrector(x, v) 
1607  
 
1608                  print 'x = ', x 
1609                  if not converged: 
1610                      if len(diag.cond) > 0: 
1611                          print "  Avg. cond. # = %lf" % (sum(diag.cond)/len(diag.cond)) 
1612                      if problem: 
1613                          # Did not converge due to failure in backward integration
 
1614                          print "  Did not converge (XB)." 
1615                          out[step-i].append(('XB', ix, x, diag)) 
1616                      elif k >= self.MaxCorrIters: 
1617                          Fnm_flag = monotone([nm[0] for nm in diag.nrm], -3,
 
1618                                              direc=-1) 
1619                          Vnm_flag = monotone([nm[1] for nm in diag.nrm], -3,
 
1620                                              direc=-1) 
1621                          cond_flag = monotone(diag.cond, -3, direc=-1) 
1622                          if Fnm_flag and Vnm_flag and cond_flag: 
1623                              # Did not converge but may have converged with more time steps
 
1624                              print "  Did not converge (XC)." 
1625                              out[step-i].append(('XC', ix, x, diag)) 
1626                          else: 
1627                              # Did not converge for unknown reason
 
1628                              print "  Did not converge (X)." 
1629                              out[step-i].append(('X', ix, x, diag)) 
1630                  else: 
1631                      print "  Converged.  Avg. cond. # = %lf" % (sum(diag.cond)/len(diag.cond)) 
1632                      # Converged
 
1633                      out[step-i].append(('C', ix, x, diag)) 
1634  
 
1635                  self._userdata.problem = False 
1636  
 
1637          grid = args() 
1638          grid.v0 = v0 
1639          grid.v1 = v1 
1640          grid.x0 = x0 
1641          grid.d0 = d0 
1642          grid.d1 = d1 
1643          grid.out = out 
1644  
 
1645          return grid

1646  
 
1647  
 


1648 -    def _curveToPointset(self):


1649          # Saves curve
 
1650          totlen = len(self.varslist + self.freepars + self.auxpars) 
1651          if totlen != self.curve.shape[1]: 
1652              # Curve of cycles
 
1653              coordnames = [] 
1654              for smtype in self.SolutionMeasures: 
1655                  coordnames += [v+'_'+smtype for v in self.varslist] 
1656              coordnames += self.freepars + self.auxpars 
1657              totlen = len(self.SolutionMeasures)*len(self.varslist) + \
 
1658                       len(self.freepars + self.auxpars) 
1659          else: 
1660              coordnames = self.varslist + self.freepars + self.auxpars 
1661          coordarray = transpose(self.curve[:self.loc+1,0:totlen]).copy() 
1662          sol = Pointset({'coordarray': coordarray,
 
1663                          'coordnames': coordnames,
 
1664                          'name': self.name}) 
1665  
 
1666          # Saves bifurcation info
 
1667          sol.labels = self.CurveInfo 
1668  
 
1669          return sol

1670  
 
1671  
 


1672 -    def getSpecialPoint(self, label1, label2=None):


1673          """Gets a point on the curve with name specified by label1 and
 
1674          label2.
 
1675  
 
1676             Inputs:
 
1677  
 
1678                 label1 -- string
 
1679                 label2 -- string
 
1680  
 
1681             Output:
 
1682  
 
1683                 x -- Point with specified name (type Point)
 
1684  
 
1685             If label2 is None, then label1 needs to be the name of the
 
1686             point.  In this case, the point type should be apparent
 
1687             from the name (i.e. by stripping off digits from the
 
1688             right).
 
1689  
 
1690             If label2 is not None, then label1 should be the point type
 
1691             and label2 the point name.
 
1692  
 
1693             For example, the following two function calls are
 
1694             equivalent:
 
1695  
 
1696                 getSpecialPoint('LP3')
 
1697                 getSpecialPoint('LP','LP3')
 
1698          """ 
1699          if label2 is None: 
1700              label2 = label1 
1701              label1 = label2.strip('0123456789') 
1702          if self.sol is not None: 
1703              ixs = self.sol.labels[label1] 
1704              l = [] 
1705              for ix in ixs: 
1706                  pt = self.sol[ix] 
1707                  if pt.labels[label1]['name'] == label2: 
1708                      l.append(pt) 
1709  #            l = [pt for pt in self.sol if pt.labels.has_key(label1) and \
 
1710  #                 pt.labels[label1].has_key('name') and \
 
1711  #                 pt.labels[label1]['name'] == label2]
 
1712          else: 
1713              raise PyDSTool_ValueError('Empty curve.  Run forward() or backward().') 
1714          if l == []: 
1715              return None 
1716          elif len(l) > 1: 
1717              raise KeyError('Ambiguous point name: More than one point with that name exists.') 
1718          else: 
1719              return l[0]

1720  
 
1721  
 


1722 -    def computeEigen(self):


1723          self.update(args(SaveEigen = True)) 
1724          ptlabel = self.curvetype.split('-')[0] 
1725          for loc, x in enumerate(self.sol): 
1726              jac = self.sysfunc.jac(x.toarray()) 
1727              jacx = jac[:,self.coords[0]:(self.coords[-1]+1)] 
1728              jacp = jac[:,self.params[0]:(self.params[-1]+1)] 
1729              w, vr = linalg.eig(jacx) 
1730              self.sol[loc].labels[ptlabel]['data'].evals = w 
1731              self.sol[loc].labels[ptlabel]['data'].evecs = vr 
1732  
 
1733              realpos = [real(eig) > 1e-6 for eig in w] 
1734              realneg = [real(eig) < -1e-6 for eig in w] 
1735              if all(realneg): 
1736                  self.sol[loc].labels[ptlabel]['stab'] = 'S' 
1737              elif all(realpos): 
1738                  self.sol[loc].labels[ptlabel]['stab'] = 'U' 
1739              else: 
1740                  self.sol[loc].labels[ptlabel]['stab'] = 'N'

1741  
 
1742  
 


1743 -    def cleanLabels(self):


1744          for pttype in all_point_types: 
1745              if pttype not in ['P', 'MX']: 
1746                  if self.sol.bylabel(pttype) is not None: 
1747                      num = 1; 
1748                      for pt in self.sol.bylabel(pttype): 
1749                          pt.labels[pttype]['name'] = pttype + repr(num) 
1750                          if pt.labels[pttype].has_key('cycle'): 
1751                              pt.labels[pttype]['cycle'].name = pttype + repr(num) 
1752                          num += 1

1753  
 
1754  
 


1755 -    def info(self):


1756          print self.__repr__() 
1757          print "Using model: %s\n"%self.model.name 
1758          if self.description is not 'None': 
1759              print 'Description' 
1760              print '----------- \n' 
1761              print self.description, '\n' 
1762          print 'Model Info' 
1763          print '---------- \n' 
1764          print "  Variables : %s"%', '.join(self.varslist) 
1765          print "  Parameters: %s\n"%', '.join(self.parsdict.keys()) 
1766          print 'Continuation Parameters' 
1767          print '----------------------- \n' 
1768          args_list = cont_args_list[:] 
1769          exclude = ['description'] 
1770          args_list.insert(2, 'auxpars') 
1771          for arg in args_list: 
1772              if hasattr(self, arg) and arg not in exclude: 
1773                  print arg, ' = ', eval('self.' + arg) 
1774          print '\n' 
1775  
 
1776          spts = '' 
1777          if self.sol is not None: 
1778              for pttype in all_point_types: 
1779                  if self.sol.bylabel(pttype) is not None: 
1780                      for pt in self.sol.bylabel(pttype): 
1781                          if pt.labels[pttype].has_key('name'): 
1782                              spts = spts + pt.labels[pttype]['name'] + ', ' 
1783          print 'Special Points' 
1784          print '-------------- \n' 
1785          print spts[:-2]

1786  
 
1787  
 


1788 -    def __repr__(self):


1789          return 'PyCont curve %s (type %s)'%(self.name,
 
1790                                          self.curvetype)

1791  
 
1792      __str__ = __repr__

1793  
 
1794  
 
1795  
 


1796 -class EquilibriumCurve(Continuation):


1797      """Child of Continuation class that represents curves of
 
1798      equilibrium points.
 
1799  
 
1800      System:
 
1801  
 
1802              h(x,a) = f(x,a) = 0
 
1803  
 
1804          The continuation variables are (x,a) with
 
1805  
 
1806              x = State variables
 
1807              a = Free parameters
 
1808  
 
1809      Detection of points: LP, H, BP""" 


1810 -    def __init__(self, model, gen, automod, plot, args=None):


1811          args['auxpars'] = [] 
1812          Continuation.__init__(self, model, gen, automod, plot, args=args) 
1813  
 
1814          self.CorrFunc = self.sysfunc

1815  
 
1816  
 


1817 -    def reset(self, args=None):


1818          """Resets curve by setting default parameters and deleting solution curve.""" 
1819          self.SPOut = None 
1820          Continuation.reset(self, args)

1821  
 
1822  
 


1823 -    def update(self, args):


1824          """Update parameters for EquilibriumCurve.""" 
1825          Continuation.update(self, args) 
1826  
 
1827          if args is not None: 
1828              for k, v in args.iteritems(): 
1829                  if k in equilibrium_args_list: 
1830                      if k == 'LocBifPoints': 
1831                          if isinstance(v, str): 
1832                              if v.lower() == 'all': 
1833                                  v = equilibrium_bif_points 
1834                              else: 
1835                                  v = [v] 
1836  
 
1837                          # Handle stopping points
 
1838                          w = [] 
1839                          if args.has_key('StopAtPoints'): 
1840                              w = args['StopAtPoints'] 
1841                              if isinstance(w, str): 
1842                                  if w.lower() == 'all': 
1843                                      w = equilibrium_bif_points 
1844                                  else: 
1845                                      w = [w] 
1846  
 
1847                          for bftype in v: 
1848                              if bftype not in cont_bif_points and \
 
1849                                 bftype not in equilibrium_bif_points: 
1850                                  if self.verbosity >= 1: 
1851                                      print "Warning: Detection of " + bftype + " points not implemented for curve EP-C." 
1852                              elif bftype == 'H' and self.varsdim == 1: 
1853                                  if self.verbosity >= 1: 
1854                                      print "Warning: Variable dimension must be larger than 1 to detect Hopf points." 
1855                              else: 
1856                                  if bftype not in self.LocBifPoints: 
1857                                      self.LocBifPoints.append(bftype) 
1858                                  if bftype in w and bftype not in self.StopAtPoints: 
1859                                      self.StopAtPoints.append(bftype) 
1860  
 
1861                      elif k != 'StopAtPoints': 
1862                          exec('self.' + k + ' = ' + repr(v))

1863  
 
1864  
 


1865 -    def _createTestFuncs(self):


1866          """Creates processors and test functions for EquilibriumCurve
 
1867          class.
 
1868  
 
1869          Note:  In the following list, processors are in
 
1870          PyCont.Bifpoint and test functions are in PyCont.TestFunc.
 
1871  
 
1872          Point type (Processor): Test function(s)
 
1873          ----------------------------------------
 
1874  
 
1875          LP (FoldPoint): Fold_Tan
 
1876                          Fold_Det
 
1877                          Fold_Bor
 
1878          H (HopfPoint): Hopf_Det
 
1879                         Hopf_Bor
 
1880          """ 
1881          Continuation._createTestFuncs(self) 
1882  
 
1883          for bftype in self.LocBifPoints: 
1884              if bftype in equilibrium_bif_points: 
1885                  stop = bftype in self.StopAtPoints  # Set stopping flag 
1886                  if bftype is 'LP': 
1887                      method1 = Fold_Bor(self.CorrFunc, self, corr=False,
 
1888                                        save=True, numpoints=self.MaxNumPoints+1) 
1889                      #method1 = Fold_Det(self.CorrFunc, self, save=True, numpoints=self.MaxNumPoints+1)
 
1890                      self.TestFuncs.append(method1) 
1891                      if 'BP' not in self.BifPoints.keys(): 
1892                          method2 = Branch_Det(self.CorrFunc, self, save=True,
 
1893                                               numpoints=self.MaxNumPoints+1) 
1894                          self.TestFuncs.append(method2) 
1895                      else: 
1896                          method2 = self.BifPoints['BP'].testfuncs[0] 
1897  
 
1898                      self.BifPoints['LP'] = FoldPoint([method1, method2],
 
1899                                                       [iszero, isnotzero],
 
1900                                                       stop=stop) 
1901                  elif bftype is 'H': 
1902                      method = Hopf_Bor(self.CorrFunc, self, corr=False,
 
1903                                        save=True, numpoints=self.MaxNumPoints+1) 
1904                      #method = Hopf_Det(self.CorrFunc, self, save=True, numpoints=self.MaxNumPoints+1)
 
1905                      #method = Hopf_Eig(self.CorrFunc, self, save=True, numpoints=self.MaxNumPoints+1)
 
1906                      self.TestFuncs.append(method) 
1907  
 
1908                      self.BifPoints['H'] = HopfPoint(method, iszero, stop=stop)

1909  
 
1910  
 
1911  
 
1912  ##############################################
 
1913  # Continuation of codimension-1 bifurcations #
 
1914  ##############################################
 
1915  
 
1916  
 


1917 -class FoldCurve(Continuation):


1918      """Child of Continuation class that represents curves of limit
 
1919      points.
 
1920  
 
1921      Augmented system h(x,a): Uses single bordering on the matrix A
 
1922      given by
 
1923  
 
1924              A = f_{x}(x,a)
 
1925  
 
1926          (see class PyCont.TestFunc.BorderMethod)
 
1927          (see class PyCont.TestFunc.Fold_Bor)
 
1928  
 
1929          The continuation variables are (x,a) with
 
1930  
 
1931              x = State variables
 
1932              a = Free parameters
 
1933  
 
1934          and the continuation curve is defined by
 
1935  
 
1936              h(x,a) = { f(x,a) = 0
 
1937                       { G(x,a) = 0
 
1938  
 
1939      Detection of points: BT, ZH, CP (BP points not currently
 
1940      implemented).
 
1941      """ 
1942  
 


1943 -    def __init__(self, model, gen, automod, plot, args=None):


1944          args['auxpars'] = [] 
1945          Continuation.__init__(self, model, gen, automod, plot, args=args) 
1946  
 
1947          self.CorrFunc = AddTestFunction(self, Fold_Bor) 
1948          self.CorrFunc.setdata(self.initpoint, None)

1949  
 
1950  
 


1951 -    def update(self, args):


1952          """Update parameters for FoldCurve.""" 
1953          Continuation.update(self, args) 
1954          if 'BP' in self.LocBifPoints: 
1955              print "BP point detection not implemented: ignoring this type of point" 
1956              self.LocBifPoints.remove('BP') 
1957          if 'BP' in self.StopAtPoints: 
1958              print "BP point detection not implemented: ignoring this type of point" 
1959              self.StopAtPoints.remove('BP') 
1960          #self.LocBifPoints = []  # Temporary fix: Delete after branch point implementation for fold curve
 
1961  
 
1962          if args is not None: 
1963              for k, v in args.iteritems(): 
1964                  if k in fold_args_list: 
1965                      if k == 'LocBifPoints': 
1966                          if isinstance(v, str): 
1967                              if v.lower() == 'all': 
1968                                  v = fold_bif_points 
1969                              else: 
1970                                  v = [v] 
1971  
 
1972                          # Handle stopping points
 
1973                          w = [] 
1974                          if args.has_key('StopAtPoints'): 
1975                              w = args['StopAtPoints'] 
1976                              if isinstance(w, str): 
1977                                  if w.lower() == 'all': 
1978                                      w = fold_bif_points 
1979                                  else: 
1980                                      w = [w] 
1981  
 
1982                          for bftype in v: 
1983                              if bftype == 'BP' or bftype not in cont_bif_points \
 
1984                                 and bftype not in fold_bif_points: 
1985                                  if self.verbosity >= 1: 
1986                                      print "Warning: Detection of " + bftype + " points not implemented for curve LP-C." 
1987                              else: 
1988                                  if bftype not in self.LocBifPoints: 
1989                                      self.LocBifPoints.append(bftype) 
1990                                  if bftype in w and \
 
1991                                     bftype not in self.StopAtPoints: 
1992                                      self.StopAtPoints.append(bftype) 
1993  
 
1994                      elif k != 'StopAtPoints': 
1995                          exec('self.' + k + ' = ' + v)

1996  
 
1997  
 


1998 -    def _createTestFuncs(self):


1999          """Creates processors and test functions for FoldCurve class.
 
2000  
 
2001          Note:  In the following list, processors are in
 
2002          PyCont.Bifpoint and test functions are in PyCont.TestFunc.
 
2003  
 
2004          Point type (Processor): Test function(s)
 
2005          ----------------------------------------
 
2006  
 
2007          BT (BTPoint): BT_Fold
 
2008          ZH (ZHPoint): Hopf_Det (or Hopf_Bor), BT_Fold
 
2009          CP (CPPoint): CP_Fold
 
2010          """ 
2011          Continuation._createTestFuncs(self) 
2012  
 
2013          for bftype in self.LocBifPoints: 
2014              if bftype in fold_bif_points: 
2015                  stop = bftype in self.StopAtPoints 
2016                  if bftype is 'BT': 
2017                      method = BT_Fold(self.CorrFunc, self, save=True,
 
2018                                       numpoints=self.MaxNumPoints+1) 
2019                      self.TestFuncs.append(method) 
2020  
 
2021                      self.BifPoints['BT'] = BTPoint(method, iszero, stop=stop) 
2022                  elif bftype is 'ZH': 
2023                      method1 = Hopf_Det(self.CorrFunc.sysfunc, self,
 
2024                                        save=True, numpoints=self.MaxNumPoints+1) 
2025                      self.TestFuncs.append(method1) 
2026                      if 'BT' not in self.BifPoints.keys(): 
2027                          method2 = BT_Fold(self.CorrFunc, self, save=True,
 
2028                                            numpoints=self.MaxNumPoints+1) 
2029                          self.TestFuncs.append(method2) 
2030                      else: 
2031                          method2 = self.BifPoints['BT'].testfuncs[0] 
2032  
 
2033                      self.BifPoints['ZH'] = ZHPoint([method1, method2],
 
2034                                                  [iszero, isnotzero], stop=stop) 
2035                  elif bftype is 'CP': 
2036                      method = CP_Fold(self.CorrFunc, self, save=True,
 
2037                                       numpoints=self.MaxNumPoints+1) 
2038                      self.TestFuncs.append(method) 
2039  
 
2040                      self.BifPoints['CP'] = CPPoint(method, iszero, stop=stop)

2041  
 
2042  
 
2043  
 


2044 -class HopfCurveOne(Continuation):


2045      """Child of Continuation class that represents curves of Hopf
 
2046      points.
 
2047  
 
2048      Augmented system h(x,a): Uses double bordering on the matrix A
 
2049      given by
 
2050  
 
2051              A = 2*f_{x}(x,a) (*) I
 
2052  
 
2053          where (*) denotes the bi-alternate matrix product.
 
2054  
 
2055          (see class PyCont.TestFunc.BiAltMethod)
 
2056          (see class PyCont.TestFunc.BorderMethod)
 
2057          (see class PyCont.TestFunc.Hopf_Double_Bor_One)
 
2058  
 
2059          The continuation variables are (x,a) with
 
2060  
 
2061              x = State variables
 
2062              a = Free parameters
 
2063  
 
2064          and the continuation curve is defined by
 
2065  
 
2066              h(x,a) = { f(x,a) = 0
 
2067                       { det(G) = 0
 
2068  
 
2069      Detection of points: BT, ZH, GH, DH
 
2070      """ 
2071  
 


2072 -    def __init__(self, model, gen, automod, plot, _args=None):


2073          _args['auxpars'] = [] 
2074          Continuation.__init__(self, model, gen, automod, plot, args=_args) 
2075  
 
2076          self.CorrFunc = AddTestFunction(self, Hopf_Double_Bor_One) 
2077          self.CorrFunc.setdata(self.initpoint, None) 
2078          self.preTF = self._bialttoeig 
2079          self.TFdata = args()

2080  
 
2081  
 


2082 -    def update(self, args):


2083          """Update parameters for HopfCurveOne.""" 
2084          Continuation.update(self, args) 
2085          if 'BP' in self.LocBifPoints: 
2086              self.LocBifPoints.remove('BP') 
2087          if 'BP' in self.StopAtPoints: 
2088              self.StopAtPoints.remove('BP') 
2089          #self.LocBifPoints = []  # Temporary fix: Delete after branch point implementation for hopf curve
 
2090  
 
2091          if args is not None: 
2092              for k, v in args.iteritems(): 
2093                  if k in hopf_args_list: 
2094                      if k == 'LocBifPoints': 
2095                          if isinstance(v, str): 
2096                              if v.lower() == 'all': 
2097                                  v = hopf_bif_points 
2098                              else: 
2099                                  v = [v] 
2100  
 
2101                          # Handle stopping points
 
2102                          w = [] 
2103                          if args.has_key('StopAtPoints'): 
2104                              w = args['StopAtPoints'] 
2105                              if isinstance(w, str): 
2106                                  if w.lower() == 'all': 
2107                                      w = hopf_bif_points 
2108                                  else: 
2109                                      w = [w] 
2110  
 
2111                          for bftype in v: 
2112                              if bftype == 'BP' or bftype not in cont_bif_points \
 
2113                                               and bftype not in hopf_bif_points: 
2114                                  if self.verbosity >= 1: 
2115                                      print "Warning: Detection of " + bftype + " points not implemented for curve H-C1." 
2116                              elif bftype == 'DH' and self.varsdim <= 3: 
2117                                  if self.verbosity >= 1: 
2118                                      print "Warning: Variable dimension must be larger than 3 to detect Double Hopf points." 
2119                              else: 
2120                                  if bftype not in self.LocBifPoints: 
2121                                      self.LocBifPoints.append(bftype) 
2122                                  if bftype in w and bftype not in self.StopAtPoints: 
2123                                      self.StopAtPoints.append(bftype) 
2124  
 
2125                      elif k != 'StopAtPoints': 
2126                          exec('self.' + k + ' = ' + repr(v))

2127  
 
2128  
 


2129 -    def _createTestFuncs(self):


2130          """Creates processors and test functions for HopfCurveOne class.
 
2131  
 
2132          Note:  In the following list, processors are in PyCont.Bifpoint
 
2133          and test functions are in PyCont.TestFunc.
 
2134  
 
2135          Point type (Processor): Test function(s)
 
2136          ----------------------------------------------
 
2137  
 
2138          BT (BTPoint): BT_Hopf_One
 
2139          DH (DHPoint): DH_Hopf
 
2140          ZH (ZHPoint): Fold_Det (or Fold_Bor)
 
2141          GH (GHPoint): GH_Hopf_One
 
2142          """ 
2143          Continuation._createTestFuncs(self) 
2144  
 
2145          for bftype in self.LocBifPoints: 
2146              if bftype in hopf_bif_points: 
2147                  stop = bftype in self.StopAtPoints  # Set stopping flag 
2148                  if bftype is 'BT': 
2149                      method = BT_Hopf_One(self.CorrFunc, self, save=True, \
 
2150                                           numpoints=self.MaxNumPoints+1) 
2151                      self.TestFuncs.append(method) 
2152  
 
2153                      self.BifPoints['BT'] = BTPoint(method, iszero, stop=stop) 
2154                  if bftype is 'DH': 
2155                      method = DH_Hopf(self.CorrFunc, self, save=True, \
 
2156                                       numpoints=self.MaxNumPoints+1) 
2157                      self.TestFuncs.append(method) 
2158  
 
2159                      self.BifPoints['DH'] = DHPoint(method, iszero, stop=stop) 
2160                  elif bftype is 'ZH': 
2161                      method = Fold_Det(self.CorrFunc.sysfunc, self, \
 
2162                                        save=True, numpoints=self.MaxNumPoints+1) 
2163                      self.TestFuncs.append(method) 
2164  
 
2165                      self.BifPoints['ZH'] = ZHPoint(method, iszero, stop=stop) 
2166                  elif bftype is 'GH': 
2167                      method = GH_Hopf_One(self.CorrFunc, self, save=True, \
 
2168                                           numpoints=self.MaxNumPoints+1) 
2169                      self.TestFuncs.append(method) 
2170  
 
2171                      self.BifPoints['GH'] = GHPoint(method, iszero, stop=stop)

2172  
 
2173  
 


2174 -    def _bialttoeig(self, X, V):


2175          q = self.CorrFunc.testfunc.data.c 
2176          p = self.CorrFunc.testfunc.data.b 
2177          n = self.sysfunc.m 
2178          A = self.sysfunc.J_coords 
2179  
 
2180          v1, v2 = invwedge(q, n) 
2181          w1, w2 = invwedge(p, n) 
2182  
 
2183          A11 = bilinearform(A,v1,v1) 
2184          A22 = bilinearform(A,v2,v2) 
2185          A12 = bilinearform(A,v1,v2) 
2186          A21 = bilinearform(A,v2,v1) 
2187          v11 = matrixmultiply(transpose(v1),v1) 
2188          v22 = matrixmultiply(transpose(v2),v2) 
2189          v12 = matrixmultiply(transpose(v1),v2) 
2190          D = v11*v22 - v12*v12 
2191          k = (A11*A22 - A12*A21)/D 
2192  
 
2193          self.TFdata.k = k[0][0] 
2194          self.TFdata.v1 = v1 
2195          self.TFdata.w1 = w1

2196  
 
2197  
 
2198  
 
2199  
 


2200 -class HopfCurveTwo(Continuation):


2201      """Child of Continuation class that represents curves of Hopf points.
 
2202  
 
2203      Augmented system h(x,a): Uses double bordering on the matrix A given by
 
2204  
 
2205              A = f_{x}^{2}(x,a) + _k*I_{n}
 
2206  
 
2207          (see class PyCont.TestFunc.BorderMethod)
 
2208          (see class PyCont.TestFunc.Hopf_Double_Bor_Two)
 
2209  
 
2210          The continuation variables are (x,a,_k) with
 
2211  
 
2212              x = State variables
 
2213              a = Free parameters
 
2214              _k = w^2 = Square of pure imaginary eigenvalue associated with Hopf point
 
2215                  (auxiliary parameter)
 
2216  
 
2217          and the continuation curve is defined by
 
2218  
 
2219                          { f(x,a) = 0
 
2220              h(x,a,_k) = { G_{1,1}(x,a,_k) = 0
 
2221                          { G_{2,2}(x,a,_k) = 0
 
2222  
 
2223      Detection of points: BT, ZH, GH
 
2224      """ 


2225 -    def __init__(self, model, gen, automod, plot, args=None):


2226          args['auxpars'] = ['_k'] 
2227          args['initpoint']['_k'] = 0 
2228          Continuation.__init__(self, model, gen, automod, plot, args=args) 
2229  
 
2230          self.CorrFunc = AddTestFunction(self, Hopf_Double_Bor_Two) 
2231  
 
2232          # Get eigenvalue information to update initpoint
 
2233          J_coords = self.sysfunc.jac(self.initpoint, self.coords) 
2234          eigs, LV, RV = linalg.eig(J_coords,left=1,right=1) 
2235          k = pow(imag(eigs[argsort(abs(real(eigs)))[0]]),2) 
2236          self.initpoint[-1] = k 
2237  
 
2238          self.CorrFunc.setdata(self.initpoint, None)

2239  
 
2240  
 


2241 -    def update(self, args):


2242          """Update parameters for HopfCurveTwo.""" 
2243          Continuation.update(self, args) 
2244          if 'BP' in self.LocBifPoints: 
2245              self.LocBifPoints.remove('BP') 
2246          if 'BP' in self.StopAtPoints: 
2247              self.StopAtPoints.remove('BP') 
2248          #self.LocBifPoints = []  # Temporary fix: Delete after branch point implementation for hopf curve
 
2249  
 
2250          if args is not None: 
2251              for k, v in args.iteritems(): 
2252                  if k in hopf_args_list: 
2253                      if k == 'LocBifPoints': 
2254                          if isinstance(v, str): 
2255                              if v.lower() == 'all': 
2256                                  v = hopf_bif_points 
2257                              else: 
2258                                  v = [v] 
2259  
 
2260                          # Handle stopping points
 
2261                          w = [] 
2262                          if args.has_key('StopAtPoints'): 
2263                              w = args['StopAtPoints'] 
2264                              if isinstance(w, str): 
2265                                  if w.lower() == 'all': 
2266                                      w = hopf_bif_points 
2267                                  else: 
2268                                      w = [w] 
2269  
 
2270                          for bftype in v: 
2271                              if bftype in ['BP', 'DH'] or bftype not in cont_bif_points and bftype not in hopf_bif_points: 
2272                                  if self.verbosity >= 1: 
2273                                      print "Warning: Detection of " + bftype + " points not implemented for curve H-C2." 
2274                              else: 
2275                                  if bftype not in self.LocBifPoints: 
2276                                      self.LocBifPoints.append(bftype) 
2277                                  if bftype in w and bftype not in self.StopAtPoints: 
2278                                      self.StopAtPoints.append(bftype) 
2279  
 
2280                      elif k != 'StopAtPoints': 
2281                          exec('self.' + k + ' = ' + repr(v))

2282  
 
2283  
 


2284 -    def _createTestFuncs(self):


2285          """Creates processors and test functions for HopfCurveTwo class.
 
2286  
 
2287          Note:  In the following list, processors are in PyCont.Bifpoint
 
2288          and test functions are in PyCont.TestFunc.
 
2289  
 
2290          Point type (Processor): Test function(s)
 
2291          ----------------------------------------------
 
2292  
 
2293          BT (BTPoint): BT_Hopf
 
2294          ZH (ZHPoint): Fold_Det (or Fold_Bor)
 
2295          GH (GHPoint): GH_Hopf
 
2296          """ 
2297          Continuation._createTestFuncs(self) 
2298  
 
2299          for bftype in self.LocBifPoints: 
2300              if bftype in hopf_bif_points: 
2301                  stop = bftype in self.StopAtPoints  # Set stopping flag 
2302                  if bftype is 'BT': 
2303                      method = BT_Hopf(self.CorrFunc, self, save=True, numpoints=self.MaxNumPoints+1) 
2304                      self.TestFuncs.append(method) 
2305  
 
2306                      self.BifPoints['BT'] = BTPoint(method, iszero, stop=stop) 
2307                  elif bftype is 'GH': 
2308                      method = GH_Hopf(self.CorrFunc, self, save=True, numpoints=self.MaxNumPoints+1) 
2309                      self.TestFuncs.append(method) 
2310  
 
2311                      self.BifPoints['GH'] = GHPoint(method, iszero, stop=stop) 
2312                  elif bftype is 'ZH': 
2313                      method = Fold_Det(self.CorrFunc.sysfunc, self, save=True, numpoints=self.MaxNumPoints+1) 
2314                      self.TestFuncs.append(method) 
2315  
 
2316                      self.BifPoints['ZH'] = ZHPoint(method, iszero, stop=stop)

2317  
 
2318  
 
2319  
 
2320  
 


2321 -class FixedPointCurve(Continuation):


2322 -    def __init__(self, model, gen, automod, plot, args=None):


2323          args['auxpars'] = [] 
2324          self.period = 1 
2325          Continuation.__init__(self, model, gen, automod, plot, args=args) 
2326  
 
2327          self._sysfunc = self.sysfunc 
2328          self.sysfunc = DiscreteMap(self._sysfunc, self.period) 
2329  
 
2330          self.CorrFunc = FixedPointMap(self.sysfunc) 
2331          self.CorrFunc.coords = self.sysfunc.coords = self.coords 
2332          self.CorrFunc.params = self.sysfunc.params = self.params

2333  
 
2334  
 


2335 -    def _preTestFunc(self, X, V):


2336          """Need CorrFunc jacobian for BP detection.""" 
2337          Continuation._preTestFunc(self, X, V) 
2338          self.CorrFunc.J_coords = self.sysfunc.J_coords - eye(self.varsdim,self.varsdim) 
2339          self.CorrFunc.J_params = self.sysfunc.J_params

2340  
 
2341  
 


2342 -    def _createTestFuncs(self):


2343          """Creates processors and test functions for EquilibriumCurve class.
 
2344  
 
2345          Note:  In the following list, processors are in PyCont.Bifpoint
 
2346          and test functions are in PyCont.TestFunc.
 
2347  
 
2348          Point type (Processor): Test function(s)
 
2349          ----------------------------------------
 
2350  
 
2351          LP (FoldPoint): Fold_Tan
 
2352                          Fold_Det
 
2353                          Fold_Bor
 
2354          H (HopfPoint): Hopf_Det
 
2355                         Hopf_Bor
 
2356          """ 
2357          Continuation._createTestFuncs(self) 
2358  
 
2359          for bftype in self.LocBifPoints: 
2360              if bftype in fixedpoint_bif_points: 
2361                  stop = bftype in self.StopAtPoints 
2362                  if bftype is 'LPC': 
2363                      method1 = LPC_Det(self.sysfunc, self, save=True, numpoints=self.MaxNumPoints+1) 
2364                      self.TestFuncs.append(method1) 
2365                      if 'BP' not in self.BifPoints.keys(): 
2366                          method2 = Branch_Det(self.CorrFunc, self, save=True, numpoints=self.MaxNumPoints+1) 
2367                          self.TestFuncs.append(method2) 
2368                      else: 
2369                          method2 = self.BifPoints['BP'].testfuncs[0] 
2370  
 
2371                      self.BifPoints['LPC'] = LPCPoint([method1, method2], [iszero, isnotzero], stop=stop) 
2372                  elif bftype is 'PD': 
2373                      method = PD_Det(self.sysfunc, self, save=True, numpoints=self.MaxNumPoints+1) 
2374                      self.TestFuncs.append(method) 
2375  
 
2376                      self.BifPoints['PD'] = PDPoint(method, iszero, stop=stop) 
2377                  elif bftype is 'NS': 
2378                      method = NS_Det(self.sysfunc, self, save=True, numpoints=self.MaxNumPoints+1) 
2379                      self.TestFuncs.append(method) 
2380  
 
2381                      self.BifPoints['NS'] = NSPoint(method, iszero, stop=stop)

2382  
 
2383  
 


2384 -    def update(self, args):


2385          """Update parameters for FixedPointCurve.""" 
2386          Continuation.update(self, args) 
2387  
 
2388          if args is not None: 
2389              for k, v in args.iteritems(): 
2390                  if k in fixedpoint_args_list: 
2391                      if k == 'LocBifPoints': 
2392                          if isinstance(v, str): 
2393                              if v.lower() == 'all': 
2394                                  v = fixedpoint_bif_points 
2395                              else: 
2396                                  v = [v] 
2397  
 
2398                          # Handle stopping points
 
2399                          w = [] 
2400                          if args.has_key('StopAtPoints'): 
2401                              w = args['StopAtPoints'] 
2402                              if isinstance(w, str): 
2403                                  if w.lower() == 'all': 
2404                                      w = fixedpoint_bif_points 
2405                                  else: 
2406                                      w = [w] 
2407  
 
2408                          for bftype in v: 
2409                              if bftype not in cont_bif_points and bftype not in fixedpoint_bif_points: 
2410                                  if self.verbosity >= 1: 
2411                                      print "Warning: Detection of " + bftype + " points not implemented for curve FP-C." 
2412                              elif bftype == 'NS' and self.varsdim == 1: 
2413                                  if self.verbosity >= 1: 
2414                                      print "Warning: Variable dimension must be larger than 1 to detect NS points." 
2415                              else: 
2416                                  if bftype not in self.LocBifPoints: 
2417                                      self.LocBifPoints.append(bftype) 
2418                                  if bftype in w and bftype not in self.StopAtPoints: 
2419                                      self.StopAtPoints.append(bftype) 
2420  
 
2421                      elif k != 'StopAtPoints': 
2422                          exec('self.' + k + ' = ' + repr(v))

2423  
 
2424  
 
2425  
 
2426  
 


2427 -class LimitCycleCurve(Continuation):


2428      """Wrapper for auto limit cycle computations.
 
2429  
 
2430      Reimplement these Continuation methods:
 
2431          _compute
 
2432          _curveToPointset
 
2433      """ 


2434 -    def __init__(self, model, gen, automod, plot, args=None):


2435          # Initialize initpoint
 
2436          args['auxpars'] = ['_T'] 
2437          args['initpoint']['_T'] = 0 
2438  
 
2439          Continuation.__init__(self, model, gen, automod, plot, args=args) 
2440  
 
2441          self.UseAuto = True 
2442          self._AdaptCycle = False 
2443  
 
2444          self.CorrFunc = self.sysfunc 
2445  
 
2446          if not hasattr(self, "NumSPOut"): 
2447              self.NumSPOut = self.MaxNumPoints 
2448  
 
2449          if not hasattr(self, "SPOut"): 
2450              self.SPOut = None

2451  
 
2452  
 


2453 -    def reset(self, args=None):


2454          """Resets curve by setting default parameters and deleting solution curve.""" 
2455          self.NumCollocation = 4 
2456          self.NumIntervals = 50 
2457          self.AdaptMesh = 3 
2458          self.DiagVerbosity = 2 
2459          self.SolutionMeasures = ['max','min'] 
2460          self.SaveFlow = False 
2461          self.SPOut = None 
2462          Continuation.reset(self, args)

2463  
 
2464  
 


2465 -    def update(self, args):


2466          """Update parameters for LimitCycleCurve.""" 
2467          Continuation.update(self, args) 
2468          if args is not None: 
2469              for k, v in args.iteritems(): 
2470                  if k in limitcycle_args_list: 
2471                      if k == 'SolutionMeasures': 
2472                          self.SolutionMeasures = ['max', 'min'] 
2473                          if isinstance(v, str): 
2474                              if v.lower() == 'all': 
2475                                  v = solution_measures.keys() 
2476                              else: 
2477                                  v = [v] 
2478  
 
2479                          for smtype in v: 
2480                              if smtype not in solution_measures.keys(): 
2481                                  if self.verbosity >= 1: 
2482                                      print "Warning: Solution measure " + smtype + " is not valid." 
2483                              elif smtype not in self.SolutionMeasures: 
2484                                  self.SolutionMeasures.append(smtype) 
2485  
 
2486                          # Order SolutionMeasures based on solution_measures
 
2487                          self.SolutionMeasures = [v for v in solution_measures_list \
 
2488                                                   if v in self.SolutionMeasures] 
2489                      elif k == 'LocBifPoints': 
2490                          if isinstance(v, str): 
2491                              if v.lower() == 'all': 
2492                                  v = limitcycle_bif_points 
2493                              else: 
2494                                  v = [v] 
2495  
 
2496                          # Handle stopping points
 
2497                          w = [] 
2498                          if args.has_key('StopAtPoints'): 
2499                              w = args['StopAtPoints'] 
2500                              if isinstance(w, str): 
2501                                  if w.lower() == 'all': 
2502                                      w = limitcycle_bif_points 
2503                                  else: 
2504                                      w = [w] 
2505  
 
2506                          for bftype in v: 
2507                              if bftype not in cont_bif_points \
 
2508                                 and bftype not in limitcycle_bif_points: 
2509                                  if self.verbosity >= 1: 
2510                                      print "Warning: Detection of %s"%bftype, 
2511                                      print " points not implemented for curve LC-C." 
2512                              else: 
2513                                  if bftype not in self.LocBifPoints: 
2514                                      self.LocBifPoints.append(bftype) 
2515                                  if bftype in w and bftype not in self.StopAtPoints: 
2516                                      self.StopAtPoints.append(bftype) 
2517                      elif k in ['NumCollocation', 'NumIntervals']: 
2518                          self._AdaptCycle = True 
2519                          exec('self.' + k + ' = ' + repr(v)) 
2520                      elif k != 'StopAtPoints': 
2521                          exec('self.' + k + ' = ' + repr(v))

2522  
 
2523  
 


2524 -    def plot_cycles(self, coords=None, cycles=None, exclude=None,
 
2525                      figure=None, axes=None, normalized=False, tlim=None,
 
2526                      method=None, color_method='default', **plot_args):


2527          if not method or method == 'highlight': 
2528              initializeDisplay(self.plot, figure=figure, axes=axes) 
2529              cfl = self.plot._cfl 
2530              cal = self.plot._cal 
2531  
 
2532          # Plot all cycles if cycles is None
 
2533          if cycles is None: 
2534              cycles = [] 
2535              for pt in self.sol: 
2536                  for ptdata in pt.labels.itervalues(): 
2537                      if ptdata.has_key('cycle'): 
2538                          cycles.append(ptdata['cycle'].name) 
2539          elif not isinstance(cycles, list): 
2540              cycles = [cycles] 
2541  
 
2542          # Initialize exclude, if necessary
 
2543          if exclude is None: 
2544              exclude = [] 
2545          elif not isinstance(exclude, list): 
2546              exclude = [exclude] 
2547  
 
2548          # Determine labeling information
 
2549          if 'label' not in plot_args: 
2550              UseCycleName = True 
2551          else: 
2552              UseCycleName = False 
2553  
 
2554          # Initialize and check coords
 
2555          if coords is None: 
2556              coords = ('t', self.varslist[0]) 
2557          for n in range(2): 
2558              if coords[n] not in ['t']+self.varslist: 
2559                  raise KeyError('Coordinate %s does not exist'%coords[n]) 
2560  
 
2561          # Get cycle pointsets from cycle names, if appropriate
 
2562          pts = [] 
2563          for cyclename in cycles: 
2564              if isinstance(cyclename, str): 
2565                  if cyclename not in exclude: 
2566                      pointtype = cyclename.strip('0123456789') 
2567                      if pointtype not in limitcycle_bif_points \
 
2568                                        + other_special_points: 
2569                          raise PyDSTool_TypeError('Wrong point type') 
2570                      cycle = self.getSpecialPoint(pointtype, cyclename) 
2571                      if cycle is None: 
2572                          raise KeyError('Cycle %s does not exist'%cyclename) 
2573                      pts.append(cycle.labels[pointtype]['cycle']) 
2574              elif isinstance(cyclename, Pointset): 
2575                  pts.append(cyclename) 
2576              else: 
2577                  print 'Point must be type(str) or type(Pointset).' 
2578          cycles = pts 
2579  
 
2580          # Get maximal period
 
2581          if coords[0] == 't': 
2582              if tlim is not None: 
2583                  if isinstance(tlim, str) and tlim[-1] == 'T' \
 
2584                              and isinstance(eval(tlim.rstrip('T')), int): 
2585                      tmult = eval(tlim.rstrip('T')) 
2586                  else: 
2587                      raise PyDSTool_TypeError("tlim must be a string of the form '#T'") 
2588              else: 
2589                  tmult = 1 
2590  
 
2591              if normalized: 
2592                  t1 = tmult 
2593              else: 
2594                  t1 = 0 
2595                  for cycle in cycles: 
2596                      #print cycle.name
 
2597                      t1 = cycle['t'][-1] > t1 and cycle['t'][-1] or t1 
2598                  t1 *= tmult 
2599  
 
2600          # Check method input
 
2601          if method is not None: 
2602              if method == 'stack' or isinstance(method, tuple) \
 
2603                        and method[0] == 'stack': 
2604                  cycperaxes = len(cycles) 
2605                  if isinstance(method, tuple): 
2606                      if len(method) == 1 or len(method) > 2: 
2607                          raise TypeError('Method should be a tuple of length 2') 
2608                      if not isinstance(method[1], int): 
2609                          raise TypeError('Need integer number of cycles') 
2610                      if method[1] < 1 or method[1] > 10: 
2611                          raise TypeError('Number of cycles per axes is limited'
 
2612                                          ' between 1 and 10') 
2613                      cycperaxes = method[1] 
2614                      method = 'stack' 
2615  
 
2616                  # Check axes input
 
2617                  if axes is None: 
2618                      axes = (1,1,[1]) 
2619                  elif isinstance(axes, pylab.Axes): 
2620                      raise TypeError('Axes must be a tuple or None') 
2621                  elif isinstance(axes[2], int): 
2622                      axes = list(axes) 
2623                      axes[2] = [axes[2]] 
2624  
 
2625                  # Initialize figure
 
2626                  initializeDisplay(self.plot, figure=figure, \
 
2627                                    axes=(axes[0], axes[1], axes[2][0])) 
2628                  figure = pylab.gcf() 
2629              elif method != 'highlight': 
2630                  raise NotImplementedError('Requested method is not implemented') 
2631  
 
2632          # Now go through cycles and plot
 
2633          for cyct, cycle in enumerate(cycles): 
2634  
 
2635              # Set up axes for 'stack' method
 
2636              if method == 'stack': 
2637                  # Get axes number and cycle number
 
2638                  axnum = int(cyct/cycperaxes) 
2639                  cycnum = cyct % cycperaxes 
2640  
 
2641                  # Handle when cycnum is 0 (i.e. new axes)
 
2642                  if cycnum == 0: 
2643                      if axnum < len(axes[2]): 
2644                          axbox = pylab.subplot(axes[0], axes[1], axes[2][axnum]) 
2645                      else: 
2646                          figure = pylab.figure() 
2647                          axbox = pylab.subplot(1,1,1) 
2648                      initializeDisplay(self.plot, figure=figure, axes=axbox) 
2649                      cfl = self.plot._cfl 
2650                      cal = self.plot._cal 
2651                      cyclenames = [] 
2652  
 
2653                  # Add cycle name to list for y labels
 
2654                  cyclenames.append(cycle.name) 
2655  
 
2656              # Initialize curve
 
2657              disp_args = copy(plot_args) 
2658              if coords[0] == 't': 
2659                  if normalized: 
2660                      numcyc = t1 
2661                      numpts = t1*len(cycle) - numcyc + 1 
2662                  else: 
2663                      #numcyc = int((t1/cycle['t'][-1]))
 
2664                      #numpts = int((t1/cycle['t'][-1])*len(cycle)) - numcyc + 1
 
2665                      numcyc = int((t1/cycle['t'][-1]))+1 
2666                      numpts = numcyc*len(cycle) - numcyc + 1 
2667                  X = zeros((2,numpts), float) 
2668                  T = normalized and cycle['t'][-1] or 1. 
2669  
 
2670                  for n in range(numcyc): 
2671                      X[0][(n*len(cycle)-n):((n+1)*len(cycle)-n)] = \
 
2672                                      (cycle['t'] + n*cycle['t'][-1])/T 
2673                      X[1][(n*len(cycle)-n):((n+1)*len(cycle)-n)] = \
 
2674                                      cycle[coords[1]] 
2675                  #X[0][(numcyc*len(cycle)-numcyc):numpts] = (numcyc*cycle['t'][-1] + cycle['t'][0:(numpts-numcyc*(len(cycle)-1))])/T
 
2676                  #X[1][(numcyc*len(cycle)-numcyc):numpts] = (cycle[coords[1]].toarray())[0:(numpts-numcyc*(len(cycle)-1))]
 
2677              else: 
2678                  X = zeros((2,len(cycle)), float) 
2679                  for n in range(2): 
2680                      if coords[n] == 't': 
2681                          X[n] = cycle['t'] 
2682                      else: 
2683                          X[n] = cycle[coords[n]] 
2684  
 
2685              # Modify curve for specified method, if necessary
 
2686              if method == 'stack': 
2687                  X[1] = (0.95/2)*X[1]/(max(abs(X[1]))*cycperaxes) + \
 
2688                         (1-(0.5+cycnum)/cycperaxes) 
2689  
 
2690              # Prints curve
 
2691              self.plot[cfl][cal][cycle.name] = pargs() 
2692              if UseCycleName: 
2693                  disp_args['label'] = cycle.name 
2694              if color_method == 'bytype' and 'c' not in disp_args and \
 
2695                                          'color' not in disp_args: 
2696                  disp_args['color'] = \
 
2697                      bif_point_colors[cycle.name.strip('0123456789')][-1] 
2698              self.plot[cfl][cal][cycle.name].cycle = \
 
2699                      pylab.plot(X[0], X[1], **disp_args) 
2700  
 
2701              # Last curve of axes was drawn, so clean up axes
 
2702              if (cyct == len(cycles)-1) or (method=='stack' and \
 
2703                                             cycnum == cycperaxes-1): 
2704                  # Handle x labels and limits
 
2705                  pylab.xlabel(coords[0]) 
2706                  if coords[0] == 't': 
2707                      pylab.xlim([0, t1]) 
2708                      if normalized: 
2709                          pylab.xlabel('Period') 
2710  
 
2711                  # Handle y labels and limits
 
2712                  if not method or method == 'highlight': 
2713                      pylab.ylabel(coords[1]) 
2714                  else: 
2715                      yticklocs = (1-(0.5+arange(cycperaxes))/cycperaxes) 
2716                      pylab.yticks(yticklocs, cyclenames) 
2717                      pylab.ylim([0, 1]) 
2718  
 
2719                  self.plot[cfl][cal].refresh() 
2720  
 
2721          # Final cleanup
 
2722          if method == 'highlight': 
2723              KeyEvent(self.plot[cfl][cal])

2724  
 
2725  
 


2726 -    def _savePointInfo(self, ds=None, evals=None, jacx=None, jacp=None,
 
2727                         flow=None, diag=None):


2728          """Created a function for this since it needs to be called
 
2729          both in _compute and when a bifurcation point is found.  It
 
2730          will have conditional statements for saving of Jacobian and
 
2731          eigenvalues, as well as other possible tidbits of information.
 
2732          """ 
2733          ptlabel = 'LC' 
2734          for ind in range(self.loc+1): 
2735              self.CurveInfo[ind] = (ptlabel, {'data': args()}) 
2736  
 
2737              if ds is not None: 
2738                  self.CurveInfo[ind][ptlabel]['data'].ds = ds[ind] 
2739  
 
2740              # Save jacobian information
 
2741              if evals is not None: 
2742                  self.CurveInfo[ind][ptlabel]['data'].evals = evals[ind] 
2743  
 
2744                  if isnan(evals[ind][0]) or abs(evals[ind][0] - 1.) > 0.05: 
2745                      # 0.05 is same tolerance used by AUTO
 
2746                      self.CurveInfo[ind][ptlabel]['stab'] = 'X' 
2747                  else: 
2748                      inside = [abs(eig) < 1. for eig in evals[ind][1:]] 
2749                      outside = [abs(eig) > 1. for eig in evals[ind][1:]] 
2750                      if all(inside): 
2751                          self.CurveInfo[ind][ptlabel]['stab'] = 'S' 
2752                      elif all(outside): 
2753                          self.CurveInfo[ind][ptlabel]['stab'] = 'U' 
2754                      elif any(isnan(inside)): 
2755                          self.CurveInfo[ind][ptlabel]['stab'] = 'X' 
2756                      else: 
2757                          self.CurveInfo[ind][ptlabel]['stab'] = 'N' 
2758  
 
2759              if jacx is not None: 
2760                  self.CurveInfo[ind][ptlabel]['data'].jac0 = \
 
2761                      reshape(jacx[0][ind], (self.varsdim, self.varsdim)) 
2762                  self.CurveInfo[ind][ptlabel]['data'].jac1 = \
 
2763                      reshape(jacx[1][ind], (self.varsdim, self.varsdim)) 
2764  
 
2765              if diag is not None: 
2766                  self.CurveInfo[ind][ptlabel]['data'].nit = diag[ind][0]

2767  
 
2768  
 


2769 -    def _compute(self, x0=None, v0=None, direc=1):


2770          """NOTE: This doesn't use v0!!!! It gets v0 from c0, which is found from x0.
 
2771          This makes sense since there can be no v0 without a c0 (v0 doesn't make sense
 
2772          for a Hopf point).""" 
2773  
 
2774          ########################
 
2775          # Initialize auto module
 
2776          ########################
 
2777          if not self._autoMod.Initialize(): 
2778              raise InitError("Initialization of auto module failed...") 
2779  
 
2780          #########################
 
2781          # Set data in auto module
 
2782          #########################
 
2783  
 
2784          # Setup index to free parameters (icp in auto)
 
2785          # BE CAREFUL OF PERIOD IN 11th SLOT!!
 
2786          freepars = [] 
2787          for P in self.freepars: 
2788              ind = self.gensys.funcspec.pars.index(P) 
2789              if ind < 10: 
2790                  freepars.append(ind) 
2791              else: 
2792                  freepars.append(ind+40) 
2793          freepars.append(10) # Add period 
2794  
 
2795          # Computation flags (special points and floguet multipliers)
 
2796          if 'PD' in self.LocBifPoints or 'NS' in self.LocBifPoints: 
2797              isp = 2 
2798          elif self.SaveEigen: 
2799              isp = 1 
2800          else: 
2801              isp = 0 
2802  
 
2803          if 'LPC' in self.LocBifPoints: 
2804              ilp = 1 
2805          else: 
2806              ilp = 0 
2807  
 
2808          # Save jacobian info
 
2809          if self.SaveJacobian: 
2810              sjac = 1; 
2811          else: 
2812              sjac = 0; 
2813  
 
2814          # Save flow info
 
2815          if self.SaveFlow: 
2816              sflow = 1; 
2817          else: 
2818              sflow = 0; 
2819  
 
2820          # Solution measures
 
2821          nsm = 1 
2822          for smtype in self.SolutionMeasures: 
2823              nsm += solution_measures[smtype] 
2824  
 
2825          # CHECK ME (Moved from below)
 
2826          parsdim = len(self.parsdict) 
2827          ipar = range(min(parsdim,10)) + [10] + range(50,parsdim+40) 
2828          parkeys = sortedDictKeys(self.parsdict) 
2829  
 
2830          # INSERT SPOut STUFF HERE
 
2831          if self.SPOut is None: 
2832              nuzr = 0 
2833              iuz = None 
2834              vuz = None 
2835          else: 
2836              iuz = [] 
2837              vuz = [] 
2838              for k, v in self.SPOut.iteritems(): 
2839                  pind = ipar[parkeys.index(k)] 
2840                  iuz.extend(len(v)*[pind]) 
2841                  vuz.extend(v) 
2842              nuzr = len(iuz) 
2843  
 
2844          self._autoMod.SetData(2,    # Problem type (ips)
 
2845                                ilp,    # No fold detection (ilp)
 
2846                                1,    # Branch switching (isw) (assuming no branch point or PD)
 
2847                                isp,    # Bifurcation point detection flag!!! (isp) 0 for now...
 
2848                                sjac,
 
2849                                sflow,
 
2850                                nsm,
 
2851                                self.MaxNumPoints,
 
2852                                self.varsdim,
 
2853                                self.NumIntervals,
 
2854                                self.NumCollocation,
 
2855                                self.AdaptMesh,
 
2856                                self.FuncTol,
 
2857                                self.VarTol,
 
2858                                self.TestTol,
 
2859                                self.MaxTestIters,
 
2860                                self.MaxCorrIters,
 
2861                                direc*self.StepSize,
 
2862                                self.MinStepSize,
 
2863                                self.MaxStepSize,
 
2864                                self.NumSPOut,
 
2865                                self.DiagVerbosity,
 
2866                                self.freeparsdim + self.auxparsdim,    # len(freepars)
 
2867                                freepars,
 
2868                                nuzr,
 
2869                                iuz,
 
2870                                vuz,
 
2871                                ) 
2872  
 
2873          #################################################
 
2874          # Set initial point in auto module (u, par, ipar)
 
2875          #################################################
 
2876          c0 = None 
2877          T = None 
2878          if x0 is None: 
2879              x0 = self.initpoint 
2880              c0 = self.initcycle 
2881  
 
2882          if isinstance(x0, dict): 
2883              # x0 is a dict on the first initial run (x0 has been parsed a little
 
2884              #   in ContClass.newCurve()
 
2885  
 
2886              # FIX ME
 
2887              x0n = x0.copy() 
2888              for k, v in x0.iteritems(): 
2889                  kn = k 
2890                  for smtype in self.SolutionMeasures: 
2891                      if k.rfind('_'+smtype) > 0: 
2892                          kn = k[0:k.rfind('_'+smtype)] 
2893                          break 
2894                  x0n[kn] = v 
2895              x0 = x0n 
2896  
 
2897              x0 = tocoords(self, x0) 
2898          elif isinstance(x0, Point): 
2899              # Check to see if point contains a cycle.  If it does, assume
 
2900              #   we are starting at a cycle and save it in initcycle
 
2901              for v in x0.labels.itervalues(): 
2902                  if v.has_key('cycle'): 
2903                      c0 = v   # Dictionary w/ cycle, name, and tangent information 
2904                      try: 
2905                          T = x0['_T'] 
2906                      except: 
2907                          T = None 
2908              x0 = x0.todict() 
2909              for p in self.freepars: 
2910                  if p not in x0.keys(): 
2911                      x0[p] = self.parsdict[p] 
2912  
 
2913              # FIX ME
 
2914              x0n = x0.copy() 
2915              for k, v in x0.iteritems(): 
2916                  kn = k 
2917                  for smtype in self.SolutionMeasures: 
2918                      if k.rfind('_'+smtype) > 0: 
2919                          kn = k[0:k.rfind('_'+smtype)] 
2920                          break 
2921                  x0n[kn] = v 
2922              x0 = x0n 
2923  
 
2924              x0 = tocoords(self, x0) 
2925  
 
2926          #u = x0.resize(x0.size())
 
2927          u = x0 
2928  
 
2929          # Set up ipars and pars, being careful with period in the 11th index
 
2930          for i, par in enumerate(self.freepars): 
2931              self.parsdict[par] = u[self.params[i]] 
2932          par = sortedDictValues(self.parsdict) 
2933  
 
2934          u = u.tolist()   # Make u a list 
2935          u.insert(0, 0.0)  # Time is in the first position 
2936  
 
2937          # Set Hopf/Cycle info in auto module
 
2938          # In particular, sets period (differently depending on Hopf vs. Cycle)
 
2939          self.CurveInfo[0] = ('P', {}) 
2940          if c0 is None: 
2941              ups = None 
2942              udotps = None 
2943              rldot = None 
2944              upslen = 0 
2945              T = CheckHopf(self, x0) 
2946          else: 
2947              t = c0['cycle']['t'] 
2948              if T is None: 
2949                  T = t[-1] - t[0] 
2950              t.resize((len(t),1)) 
2951              t = (t-t[0])/T 
2952  
 
2953              v = transpose(c0['cycle'].toarray()) 
2954              ups = array(c_[t, v]) 
2955              upslen = len(ups) 
2956              ups = resize(ups, ups.size) 
2957              ups = ups.tolist()  # One dim list 
2958  
 
2959              udotps = c0['data'].V['udotps'] 
2960              if udotps is not None: 
2961                  udotps = resize(udotps, udotps.size) 
2962                  udotps = udotps.tolist()    # One dim list 
2963  
 
2964              rldot = c0['data'].V['rldot'] 
2965              if rldot is not None: 
2966                  rldot = c0['data'].V['rldot'].tolist() 
2967  
 
2968          # Insert period T in par and u list.
 
2969          if T is not None: 
2970              par.insert(10, T) 
2971          else: 
2972              if not self._autoMod.ClearAll(): 
2973                  raise RuntimeError('Cleanup of auto module failed...') 
2974              raise PyDSTool_ExistError("Period missing in starting point... aborting.") 
2975  
 
2976          # Load data into auto module and call AUTO if successful
 
2977          #print ups
 
2978          if self._autoMod.SetInitPoint(u[0:self.varsdim+1],parsdim+1,ipar,par,
 
2979                         freepars, upslen, ups, udotps, rldot, self._AdaptCycle): 
2980              AutoOut = self._autoMod.Compute() 
2981  
 
2982              ##############
 
2983              # Unpack stuff
 
2984              ##############
 
2985  
 
2986              # Save curve (all solution measures and parameters at end)
 
2987              self.curve = c_[AutoOut[0], AutoOut[1]] 
2988              for i in range(len(self.SolutionMeasures)-1): 
2989                  self.curve = c_[self.curve, AutoOut[2+i]] 
2990              self.loc = len(self.curve)-1    # This might change based on bad points 
2991              badpts = nonzero([any(isnan(pt)) for pt in self.curve])[0] 
2992              if len(badpts) > 0: 
2993                  # This needs to be changed to the special point just previous to this
 
2994                  endpt = badpts[0]-1 
2995              else: 
2996                  endpt = self.loc 
2997  
 
2998              # TEMP: Save floquet multipliers
 
2999              extra_out = 0 
3000              evals = None 
3001              if self.SaveEigen or 'PD' in self.LocBifPoints or \
 
3002                                   'NS' in self.LocBifPoints: 
3003                  self.SaveEigen = True 
3004                  evals = AutoOut[1+len(self.SolutionMeasures)] 
3005                  extra_out += 1 
3006  
 
3007              # Jacobian info
 
3008              jacx = None 
3009              if self.SaveJacobian: 
3010                  jacx = (AutoOut[1+len(self.SolutionMeasures)+extra_out], \
 
3011                          AutoOut[1+len(self.SolutionMeasures)+extra_out+1]) 
3012                  extra_out += 2 
3013  
 
3014              # Save number of iterations
 
3015              nit = None 
3016              if 1: 
3017                  nit = AutoOut[1+len(self.SolutionMeasures)+extra_out] 
3018                  extra_out += 1 
3019  
 
3020              # Insert cycle info and determine stopping point
 
3021              sp_endpt = 0 
3022              sp_endpt_type = 'P' 
3023              for i in range(1+len(self.SolutionMeasures)+extra_out, len(AutoOut)): 
3024                  (sp_ind, sp_type, sp_ups, sp_udotps, sp_rldot, sp_flow) = AutoOut[i] 
3025                  if sp_ups is not None and sp_ind <= endpt: 
3026                      pttype = auto_point_types[sp_type] 
3027  
 
3028                      # Only save those that are asked for
 
3029                      # (auto calculates PD and NS together)
 
3030                      if pttype in self.LocBifPoints + other_special_points: 
3031                          sp_endpt = sp_ind 
3032                          sp_endpt_type = pttype 
3033  
 
3034                          # Scale time to period
 
3035                          sp_ups[:,0] *= self.curve[sp_ind][-1] 
3036  
 
3037                          # Change cycle to parametrized Pointset
 
3038                          # (MAY WANT TO GENERALIZE _curveToPointset!)
 
3039                          coordnames = self.varslist 
3040                          coordarray = transpose(sp_ups[:,1:]).copy() 
3041                          indepvarname = 't' 
3042                          indepvararray = transpose(sp_ups[:,0]).copy() 
3043                          sp_ups = Pointset({'coordarray': coordarray,
 
3044                                             'coordnames': coordnames,
 
3045                                             'indepvarname': indepvarname,
 
3046                                             'indepvararray': indepvararray}) 
3047  
 
3048                          self.CurveInfo[sp_ind] = (pttype, \
 
3049                              {'cycle': sp_ups, 'data': args(V = \
 
3050                                  {'udotps': sp_udotps, 'rldot': sp_rldot})}) 
3051  
 
3052                          # Save flow
 
3053                          if sp_flow is not None: 
3054                              self.CurveInfo[sp_ind][pttype]['flow'] = sp_flow 
3055  
 
3056              if sp_endpt < self.loc: 
3057                  if self.verbosity > 0: 
3058                      print 'Warning: NaNs in solution from AUTO. ', 
3059                      print 'Reduce stepsize and try again.' 
3060                  self.loc = sp_endpt 
3061                  self.curve = self.curve[0:self.loc+1] 
3062                  self.CurveInfo[self.loc] = ('MX', \
 
3063                                      self.CurveInfo[self.loc][sp_endpt_type]) 
3064                  self.CurveInfo.remove(self.loc, sp_endpt_type) 
3065  
 
3066              # Initialize labels
 
3067              self._savePointInfo(evals=evals, jacx=jacx, diag=nit) 
3068              #self.CurveInfo[i] = ('LC', {})
 
3069  
 
3070              self._AdaptCycle = False 
3071          else: 
3072              if not self._autoMod.ClearAll(): 
3073                  raise RuntimeError('Cleanup of auto module failed...') 
3074              raise PyDSTool_ExistError('Bad initial point/cycle.  No curve computed.') 
3075          ### END CALL TO AUTO
 
3076  
 
3077          #########
 
3078          # Cleanup
 
3079          #########
 
3080          if not self._autoMod.ClearAll(): 
3081              raise RuntimeError('Cleanup of auto module failed...')

3082  
 
3083  
 


3084 -    def info(self):


3085          Continuation.info(self) 
3086  
 
3087          print '\nLimit Cycle Curve Parameters' 
3088          print '------------------------------\n' 
3089          args_list = limitcycle_args_list[:] 
3090          args_list.remove('LocBifPoints') 
3091          for arg in args_list: 
3092              if hasattr(self, arg): 
3093                  print arg, ' = ', eval('self.' + arg) 
3094          print '\n'

3095  
 
3096  
 
3097  
 
3098  
 


3099 -class UserDefinedCurve(Continuation):


3100      """User defined curve.  We'll see how this goes...""" 
3101  
 


3102 -    def __init__(self, model, gen, automod, plot, initargs=None):


3103          initargs['auxpars'] = [] 
3104  
 
3105          # Initialize user information
 
3106          self.varslist = initargs['uservars'] 
3107          self.parsdict = initargs['userpars'].copy() 
3108          self._userfunc = initargs['userfunc'] 
3109          self._userdata = args() 
3110          if initargs.has_key('userjac'): 
3111              self._userjac = initargs['userjac'] 
3112          if initargs.has_key('usertestfuncs'): 
3113              self._usertestfuncs = initargs['usertestfuncs'] 
3114          if initargs.has_key('userbifpoints'): 
3115              self._userbifpoints = initargs['userbifpoints'] 
3116          else: 
3117              self._userbifpoints = [] 
3118          if initargs.has_key('userdomain'): 
3119              self._userdomain = initargs['userdomain'] 
3120  
 
3121          [initargs.pop(i) for i in ['uservars','userpars','userjac','userfunc',
 
3122                  'usertestfuncs','userbifpoints','userdomain'] if i in initargs] 
3123          Continuation.__init__(self, model, gen, automod, plot, args=initargs) 
3124  
 
3125          self.CorrFunc = self.sysfunc

3126  
 
3127  
 


3128 -    def update(self, args):


3129          """Update parameters for UserDefinedCurve.""" 
3130  
 
3131          Continuation.update(self, args) 
3132          if args is not None: 
3133              for k, v in args.iteritems(): 
3134                  if k in userdefined_args_list: 
3135                      exec('self.' + k + ' = ' + repr(v))

3136  
 
3137  
 


3138 -    def _createTestFuncs(self):


3139          """Creates processors and test functions for UserDefinedCurve class.
 
3140  
 
3141          Note:  In the following list, processors are in PyCont.Bifpoint
 
3142          and test functions are in PyCont.TestFunc.
 
3143  
 
3144          Point type (Processor): Test function(s)
 
3145          ----------------------------------------
 
3146  
 
3147          LP (FoldPoint): Fold_Tan
 
3148                          Fold_Det
 
3149                          Fold_Bor
 
3150          H (HopfPoint): Hopf_Det
 
3151                         Hopf_Bor
 
3152          """ 
3153          Continuation._createTestFuncs(self) 
3154  
 
3155          for bftype in self.LocBifPoints: 
3156              stop = bftype in self.StopAtPoints  # Set stopping flag 
3157  
 
3158              if bftype in self._userbifpoints: 
3159                  tfuncs = [] 
3160                  tflags = [] 
3161                  if not isinstance(self._userbifpoints[bftype], list): 
3162                      self._userbifpoints[bftype] = [self._userbifpoints[bftype]] 
3163                  for tfunc in self._userbifpoints[bftype]: 
3164                      tfunc_name = tfunc[0] 
3165                      tfunc_flag = tfunc[1] 
3166                      if tfunc_flag == 0: 
3167                          tflags.append(iszero) 
3168                      else: 
3169                          tflags.append(isnotzero) 
3170                      tfunc_func = self._usertestfuncs[tfunc_name][0] 
3171                      tfunc_dim = self._usertestfuncs[tfunc_name][1] 
3172  
 
3173                      method = UserDefinedTestFunc((self.sysfunc.n, tfunc_dim),
 
3174                                                  self, tfunc_func, save=True,
 
3175                                                  numpoints=self.MaxNumPoints+1) 
3176                      self.TestFuncs.append(method) 
3177                      tfuncs.append(method) 
3178  
 
3179                  self.BifPoints[bftype] = BifPoint(tfuncs, tflags,
 
3180                                                    label=bftype, stop=stop)

3181
```

  


| Home | Trees | Indices | Help | PyDSTool |
| --- | --- | --- | --- | --- |

|  |  |
| --- | --- |
| Generated by Epydoc 3.0.1 on Fri May 4 15:24:21 2012 | http://epydoc.sourceforge.net |
